# Test-retest reliability of the human functional connectome over consecutive days: identifying highly reliable portions and assessing the impact of methodological choices

**DOI:** 10.1162/netn_a_00148

**Published:** 2020-09-01

**Authors:** Leonardo Tozzi, Scott L. Fleming, Zachary D. Taylor, Cooper D. Raterink, Leanne M. Williams

**Affiliations:** Department of Psychiatry and Behavioral Sciences, Stanford University, Stanford, CA, USA; Department of Biomedical Data Science, Stanford University, Stanford, CA, USA; Department of Computer Science, Stanford University, Stanford, CA 94305, USA; Department of Computer Science, Stanford University, Stanford, CA 94305, USA; Department of Psychiatry and Behavioral Sciences, Stanford University, Stanford, CA, USA

**Keywords:** fMRI, Functional connectivity, Resting state, Reliability

## Abstract

Countless studies have advanced our understanding of the human brain and its organization by using functional magnetic resonance imaging (fMRI) to derive network representations of human brain function. However, we do not know to what extent these “functional connectomes” are reliable over time. In a large public sample of healthy participants (*N* = 833) scanned on two consecutive days, we assessed the test-retest reliability of fMRI functional connectivity and the consequences on reliability of three common sources of variation in analysis workflows: atlas choice, global signal regression, and thresholding. By adopting the intraclass correlation coefficient as a metric, we demonstrate that only a small portion of the functional connectome is characterized by good (6–8%) to excellent (0.08–0.14%) reliability. Connectivity between prefrontal, parietal, and temporal areas is especially reliable, but also average connectivity within known networks has good reliability. In general, while unreliable edges are weak, reliable edges are not necessarily strong. Methodologically, reliability of edges varies between atlases, global signal regression decreases reliability for networks and most edges (but increases it for some), and thresholding based on connection strength reduces reliability. Focusing on the reliable portion of the connectome could help quantify brain trait-like features and investigate individual differences using functional neuroimaging.

## Author Summary

We quantified the reliability of fMRI functional connectivity in a large sample of healthy participants (*N* = 833) scanned over two consecutive days. We also assessed the consequences on reliability of atlas choice, global signal regression and thresholding. Only a small portion of the functional connectome has good (6–8%) to excellent (0.08–0.14%) reliability. Connectivity between prefrontal, parietal and temporal areas is especially reliable and average connectivity within known networks has good reliability. While unreliable edges are generally weak, reliable edges are not necessarily strong. Reliability of edges varies between atlases. Global signal regression decreases reliability for networks and most edges (but increases it for some). Thresholding based on connection strength reduces reliability. Focusing on reliable portions of the connectome could help investigate individual differences using functional neuroimaging.

## INTRODUCTION

The human brain is an extraordinarily complex network comprising one hundred billion neurons, each connected to an average of 7,000 other neurons. This yields between 100 trillion and 1 quadrillion synapses, depending on a person’s age (Drachman, 2005). Current research in neuroscience suggests that it is the architecture and dynamic interactions of neurons that give rise to complex phenomena, such as cognition and emotion (Bassett & Sporns, 2017; Lindquist et al., 2012; Mill et al., 2017). This has been called the “functional connectome,” and over the past three decades, several studies have characterized it in vivo (see, for example, Van Essen et al., 2013). There is consensus that we need to map the human brain connectome in order to move forward our fundamental understanding of the human brain and its organization. However, we do not yet know to what extent the functional connectome is stable over time for an individual. In the present work, our aim is to explore the short-term reliability (in the order of days) of functional connectomes. We believe this is a fundamental step toward the identification of a persistent representation of brain function, which will facilitate the mapping of cognitive processes in individuals and will be critical for linking connectivity disruptions to brain disorders.

Over the past three decades, functional connectivity (FC) has become a well-established approach to measuring the functional connectome by using functional magnetic resonance imaging (fMRI). The term FC refers to synchronous distributed fluctuations in neuronal activity and is thought to represent a correlate of the dynamic interaction between neurons located in different brain areas (Lowe et al., 2000). In most studies, FC is measured by computing a Pearson correlation of the fMRI-derived blood oxygen level–dependent (BOLD) time series of a set of regions while the participant is awake and not performing any task (Lowe et al., 2016). This is what we will intend as FC in the present work, but it is important to note that FC can also be computed while participants are not completely idle and by using a wide array of methods besides correlations, such as independent component analysis, analyses in the frequency domain, Bayesian models, and dynamic approaches (for review, Gonzalez-Castillo & Bandettini, 2018; Lowe et al., 2016; Preti et al., 2017; Smitha et al., 2017).

In recent years, FC has been used to answer important questions about both healthy and disordered brain function. For example, psychiatry and neurology have turned to FC to develop new diagnostic tests, predict treatment response, and relate brain function to symptoms (Fornito et al., 2015; Sha et al., 2018). This is in answer to an urgent need for quantitative correlates of brain illnesses, and network-based approaches show great promise to this end (Williams, 2017). However, when testing candidate measures for clinical applications, it is important to consider that measures of robust group-level effects, which make up a large portion of the existent literature, might not necessarily be suited to make inferences about individuals (Hedge et al., 2018). For example, a network might show consistent FC values because of its low between-subjects variability, but this same characteristic would make it unsuitable to investigate its correlations with measures that might be highly variable between subjects (symptoms, personality, etc.). Also, one reasonable characteristic of a measure considered for clinical applications is repeatability: the same test on the same individual after a short period of time should return very similar results (Sullivan et al., 2015). In the present work, we define reliability as the combination of high between-subjects variability, coupled with low within-subject variability, a combination that is advantageous when trying to relate biomarkers to individual traits (Fleiss et al., 2003; Zuo et al., 2019b). Unfortunately, several of the measurements commonly collected by researchers to relate brain function to behavior have poor reliability (Hedge et al., 2018), which might explain their weak relationship to trait-like features (see, for example, Eisenberg et al., 2019). It follows that for any study investigating individual differences using neuroimaging, ensuring the reliability of measurements is of paramount importance, even outside of the realm of clinical applications (Xing & Zuo, 2018; Zuo et al., 2019a, 2019b).

To date, only a relatively small number of studies has examined in detail the reliability of the functional connectome, summarized by two recent papers (Noble et al., 2019; Zuo & Xing, 2014).There is consensus that connectomes tend to become more reliable the longer the duration of the scan (Anderson et al., 2011; Birn et al., 2013; Elliott et al., 2019; Noble et al., 2019; Termenon et al., 2016). However, which and how many functional connections can be measured reliably is unclear. Meta-analytical evidence shows that, on average, FC reliability is poor (Noble et al., 2019). Some studies, however, report that functional connections have “fair” reliability on average (as defined by Cicchetti, 1994) but others report “good” or “excellent” reliability for large (>25%) portions of the functional connectome, in particular of well-characterized functional networks such as the default mode, fronto-parietal, and dorsal attention networks (Birn et al., 2013; Elliott et al., 2019; Guo et al., 2012; Zuo & Xing, 2014). Reliability of global and local graph metrics derived from functional connectomes has also been shown to only be in the “fair” range, but can still be considered statistically significant (Termenon et al., 2016). One reason for these discrepancies might be that most studies only used relatively small samples (*N* <= 50) and had long intervals between scanning sessions (days or months). Sample size has been found to affect reliability estimates (Termenon et al., 2016), and shorter time intervals are better suited for measuring test-retest reliability, since they minimize the variability introduced by ancillary factors (Sullivan et al., 2015). Also, there are several choices that are routinely made when computing functional connectomes and it is unclear how these might affect their reliability. Examining all such possible choices is beyond the possibilities of the current work, but we focus here on three procedures for which a clear guideline in the community is lacking. The first is the set of regions (atlas) used to compute FC. Choosing atlases with a higher number of regions has been shown to increase reliability (Termenon et al., 2016), but it is unclear if a consensual pattern of reliable connections exists across atlases. Second, global signal regression term (GSR) is often used as a denoising procedure but potentially affects FC and has been suggested to decrease reliability (Elliott et al., 2019; Murphy & Fox, 2017; Power et al., 2018). Third, to compute graph metrics based on the functional connectome that require sparsity, usually weaker connections are removed, either based on an absolute or relative threshold, that is, edges below a certain value, or the bottom *n*^th^ percentile. Different graph metrics appear to have highest reliability at different thresholds (Termenon et al., 2016). It is, however, unclear if edge strength and reliability are related, how thresholding affects the reliability of edges, and even if the same edges are retained when functional connectomes from the same individual are thresholded independently.

In the present work, we explore short-term test-retest reliability of functional connectomes in a very large sample of healthy individuals scanned on two consecutive days. In particular, we leverage the entire Human Connectome Project (HCP) Healthy Young Adult data release. This dataset makes use of cutting-edge acquisition and preprocessing techniques, has very long resting-state sessions (30 min), is publicly available, and is a widely used gold standard for transparent and reproducible methods testing. In these ideal conditions, first, we assess reliability of the edges and known networks that make up the functional connectome. Then, we examine the impact on reliability of atlas choice, GSR, and thresholding.

## MATERIALS AND METHODS

All scripts to reproduce our analyses and plots are available on GitHub at https://github.com/leotozzi88/reliability_study. The data processing flow is shown in Supporting Information Figure S1, along with the name of the scripts in this repository corresponding to each analysis step.

### Dataset

Our sample is derived from the HCP Healthy Young Adult release, a large public dataset of 1,200 subjects aged between 22 and 35 years without any psychiatric or neurological disorder (Van Essen et al., 2013). The acquisition parameters and minimal preprocessing of these data are described in Glasser et al. (2013). Briefly, participants underwent a large number of MRI scans, that included T1- and T2-weighted structural imaging, diffusion tensor imaging, and nearly 2 hours of resting-state and task multiband fMRI. For the present study, we used 1 hour of resting-state fMRI collected on each participant during four 15-min scans (1,200 time points each, two runs acquired with RL phase encoding and two with LR) split in two scanning sessions over two days.

To select our sample, we accessed the data at https://db.humanconnectome.org. Using the online filtering options, we selected only participants who had completed the full resting-state scanning protocol and had no known quality issues. This returned a total of 860 subjects, each with four resting-state fMRI runs. For these, we downloaded the framewise relative root-mean-square realignment estimates (RMS) and fMRI data denoised using ICA-FIX (Salimi-Khorshidi et al., 2014). All analyses were conducted in greyordinate space, that is, they were constrained to the gray matter by using files in the CIFTI format, thus taking full advantage of HCP preprocessing and minimizing nonneuronal signal (Glasser et al., 2013).

### Connectivity Matrix Construction

Each dense denoised timeseries resting-state file was parcellated using connectome workbench (wb_command-cifti-parcellate) to obtain the mean timeseries in each atlas region (see below). All further analyses were conducted in MATLAB R2018a (9.4.0.949201) for Mac (The MathWorks, Inc.). For each subject, parcellated timeseries as well as framewise RMS were loaded. Then, GSR was performed (see below). A high-pass filter (0.008-Hz cut-off) was applied to the timeseries. High frequencies were retained to avoid excessive loss of degrees of freedom due to the very low TR (Bright et al., 2017). Volumes with RMS > 0.30 were flagged as containing motion and were removed from the timeseries (Power et al., 2014). We also excluded any subject for which volumes flagged for motion exceeded 15% in any of the four resting-state runs. This step resulted in a final sample size of 833. The two runs within each one of the two sessions were then concatenated, and Pearson correlation between all the timeseries was used to obtain a connectivity matrix for each session. At the end of this procedure, each subject had six matrices (3 atlases, with and without GSR) for each of two sessions, for a total of 9,996 connectivity matrices.

### Connectivity of Established Resting-State Networks

From each connectivity matrix, the average FC within each of 12 established resting-state networks was computed using the labels of the Gordon Atlas (Gordon et al., 2016). These resting-state networks were default mode, parieto-occipital, fronto-parietal, salience, cingulo-opercular, medial-parietal, dorsal attention, ventral attention, visual, supplementary motor (hand), supplementary motor (mouth), and auditory. The reliability of these aggregate network statistics was then assessed.

### Test-Retest Reliability

Intraclass correlation coefficient (ICC) as implemented in MATLAB (https://www.mathworks.com/matlabcentral/fileexchange/22099-intraclass-correlation-coefficient-icc) was used to quantify the test-retest reliability of FC. In particular, we assessed the consistency among measurements under the fixed levels of the session factor, in line with previous work (Chen et al., 2018; Elliott et al., 2019). This measure has been named ICC ‘C-1’ in McGraw and Wong (1996) or ICC (3,1) in Shrout and Fleiss (1979).

To calculate ICC for all our FC values, first, the FC values in the upper triangle of each subject’s connectivity matrix were entered as rows in two large matrices (one matrix for each session, one row per subject in each matrix). Then, the corresponding columns of these matrices were compared to obtain an ICC value. Since the number of features (and thus ICCs) was very large (from 21,945 to 61,776 depending on atlas), we report the median, minimum, and maximum ICC as well as the portion of functional connections having poor (<0.40), fair (0.40–0.60), good (0.60–0.75), or excellent (>0.75) ICC as defined by Cicchetti (1994).

This procedure was conducted for connectivity matrices obtained using all atlases, with and without GSR as well as for the resting-state networks average FC.

### Effects of Atlas on Reliability

For each subject and each session, we computed functional connectomes by using three atlases that are widely used within the neuroimaging community and available in CIFTI format. The first is the Brainnettome Atlas, derived by structural and FC (Fan et al., 2016). The second is the Glasser Atlas, based on the multimodal cortical parcellation of HCP participants (Glasser et al., 2016).The third is the Gordon Atlas, which is based on resting-state FC and provides labels identifying well-established resting-state networks (Gordon et al., 2016). Since none of three atlases includes subcortical structures, these were derived from the Freesurfer segmentation (Fischl et al., 2002) and added to each CIFTI dense label file using connectome workbench (wb_command-cifti-create-dense-from-template). To test for a difference in ICC values across atlases, we used a Kruskal–Wallis test.

### Effect of Global Signal Regression on Reliability

Immediately after loading the timeseries data in MATLAB, the mean of the grayordinate timeseries from all regions was regressed from each timeseries to produce a set of GSR-corrected timeseries (Burgess et al., 2016). Analyses then proceeded in the same way separately for GSR-corrected (GSR+) and noncorrected (GSR−) timeseries. To test for a difference in ICC values computed using GSR+ and GSR− timeseries, we used a Wilcoxon signed rank test.

### Effect of Edge Strength on Reliability

To get a measure of edge strength, we averaged the FC of all edges across the two sessions and across all subjects. Then, for each atlas, we computed a Spearman correlation between edge strength and ICC of the edge calculated as outlined above.

### Effects of Thresholding on Reliability

To test the effects of thresholding the functional connectome on reliability estimates and if the same edges would be consistently retained across sessions, we proceeded as follows. First, we defined 20 evenly spaced threshold values from 0.05 to 1. For each value and each connectivity matrix, two new matrices were created using functions from the brain connectivity toolbox (Rubinov & Sporns, 2010). In the first matrix, all FC values below the threshold were set to 0 (absolute threshold). In the second, the proportion of strongest FC values corresponding to the threshold was retained (relative threshold). Then, for each absolute and relative threshold, we examined all edges that were retained at least in one session. We computed the ratio between the number of participants in which each edge was retained at both time points versus the ones in which it was retained at least once. This quantity, which we name “ratio of consistent edges,” or “consistency ratio” for short, goes from 0 (each time the edge is retained, it is only retained in one session) to 1 (each time the edge is retained, it is retained in both sessions). We report the median, minimum, and maximum ratio of consistent edges for each threshold and atlas as well as the proportion of poor (<0.40), fair (0.40–0.60), good (0.60–0.75), or excellent (>0.75) edges using the same cutoffs as for ICC for convenience (Cicchetti, 1994). We also tested whether the median ratio of consistent edges was correlated with the threshold value by using a Spearman correlation. Finally, for each absolute and relative threshold, we computed the ICC as described above only in subjects for which the edge was retained in both sessions, so as not to bias our calculation by the edge potentially being set to 0 because of thresholding in one session. We also computed whether median ICC values were correlated with the threshold value by using a Spearman correlation.

### Confirmation of Results in Nonrelated Subsample

Since several subjects in the Healthy Young Adult dataset share family membership and a significant portion of variance in FC is explained by genetics (Adhikari et al., 2018; Elliott et al., 2019; Reineberg et al., 2018), we reran our analyses on an unrelated subset of our dataset to confirm our results (*N* = 397 after accounting for data availability and motion).

### Confirmation of Results Using Different ICC Intervals

Since the cutoffs reported in Cicchetti (1994) are just one of the possible ways to classify ICC values, we reran our analyses using a more fine-grained binning scheme with five instead of four classes: slight (<0.20), fair (0.20–0.40), moderate (0.40–0.60), substantial (0.60–0.80), and perfect (>0.80) (Xing & Zuo, 2018).

## RESULTS

### Test-Retest Reliability of the Functional Connectome

**Figure 1.**
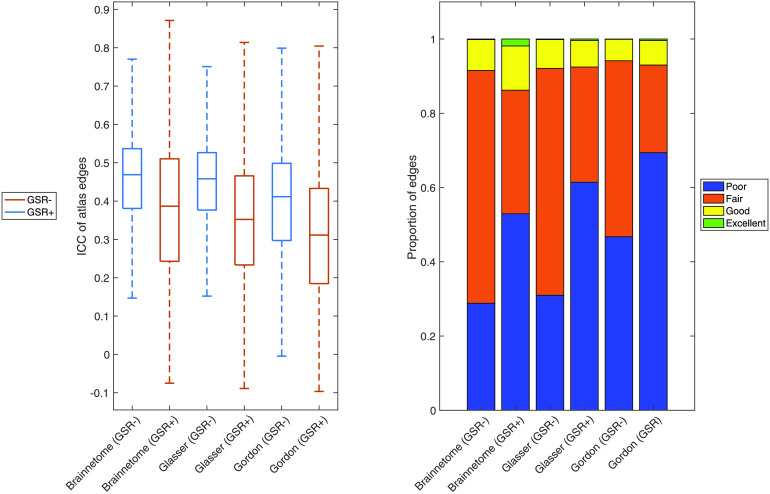
Reliability of functional connectome edges. Left: For the Brainnetome, Glasser, and Gordon atlases with and without performing GSR, we show boxplots of ICC of all atlas edges. Right: For the Brainnetome, Glasser, and Gordon atlases with and without performing GSR, we show the proportion of edges having poor (ICC < 0.40), fair (ICC = 0.40–0.60), good (ICC = 0.60–0.75), or excellent (ICC > 0.75) reliability, defined in accordance to Cicchetti (1994). GSR− = no global signal regression; GSR+ = global signal regression; ICC = intraclass correlation coefficient.

Regardless of atlas and without performing GSR (see below), the majority of edges of the functional connectome were in the “fair” reliability range (Figure 1).

**Figure 2.**
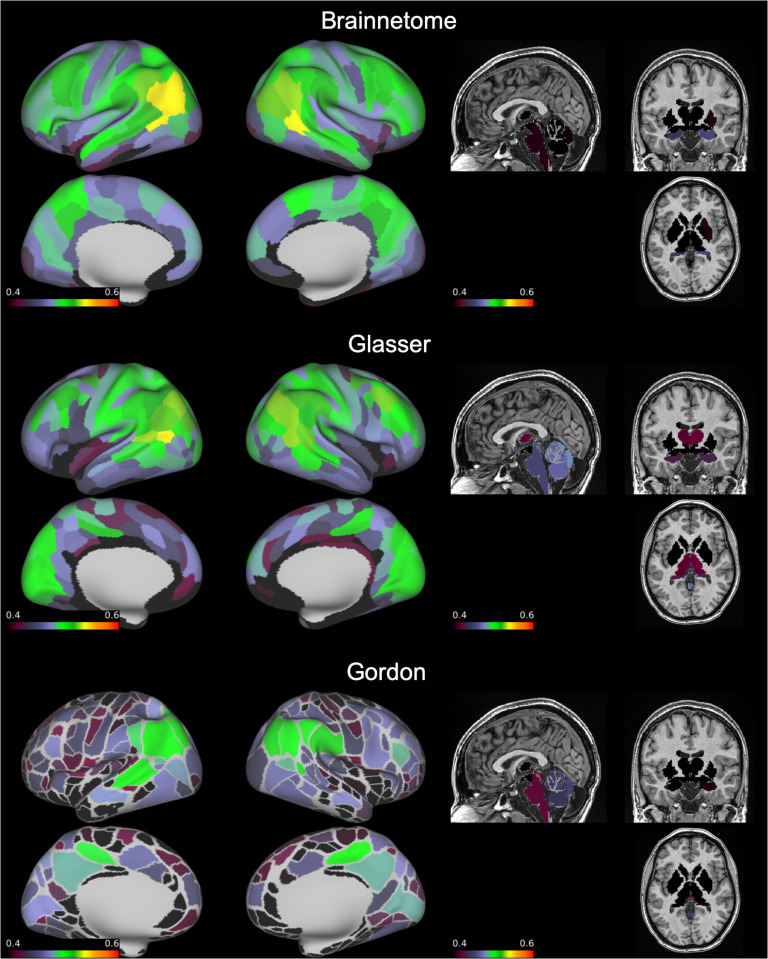
Average ICC of functional connections by node. On an inflated brain we show, for each node of the Brainnetome, Glasser, and Gordon atlases, the average ICC of all functional connections involving the node. ICC = intraclass correlation coefficient.

Median ICC ranged from 0.41 to 0.47 depending on atlas (Table 1 and Figure 1). Given the large sample size, estimation of ICC was accurate: the width of confidence intervals for ICC in all three atlases varied between 0.04 and 0.14 and had a median of 0.10 (Supporting Information Figure S2). When examining the average ICC of all edges touching each node, the subgenual anterior cingulate cortex and inferior temporal lobe had the lowest average reliability. Average ICC was also low in areas immediately adjacent to the corpus callosum (cingulate cortex). The most reliable nodes on average were located in the superior parietal and middle temporal lobes (Figure 2).

**Table 1.** Reliability of edges in the functional connectome.

		**Median ICC**	**Min ICC**	**Max ICC**	**Poor edges ratio**	**Fair edges ratio**	**Good edges ratio**	**Excellent edges ratio**
**Brainnetome**	GSR−	0.4688	−0.0682	0.8145	0.2884	0.6271	0.0832	0.0014
	GSR+	0.3868	−0.0750	0.8713	0.5294	0.3328	0.1191	0.0187
**Glasser**	GSR−	0.4587	−0.0627	0.8277	0.3085	0.6124	0.0777	0.0014
	GSR+	0.3522	−0.0889	0.8643	0.6143	0.3103	0.0714	0.0040
**Gordon**	GSR−	0.4122	−0.0845	0.8102	0.4663	0.4755	0.0574	0.0008
	GSR+	0.3113	−0.0965	0.8453	0.6939	0.2361	0.0663	0.0037

*Note*. We show the median, minimum, and maximum ICC of functional connectomes computed using three different atlases, with or without global signal regression. We also show the proportion of edges having poor (ICC < 0.40), fair (ICC = 0.40–0.60), good (ICC = 0.60–0.75), or excellent (ICC > 0.75) reliability, defined in accordance to Cicchetti (1994). ICC = intraclass correlation coefficient; GSR− = no global signal regression; GSR+ = global signal regression.

**Figure 3.**
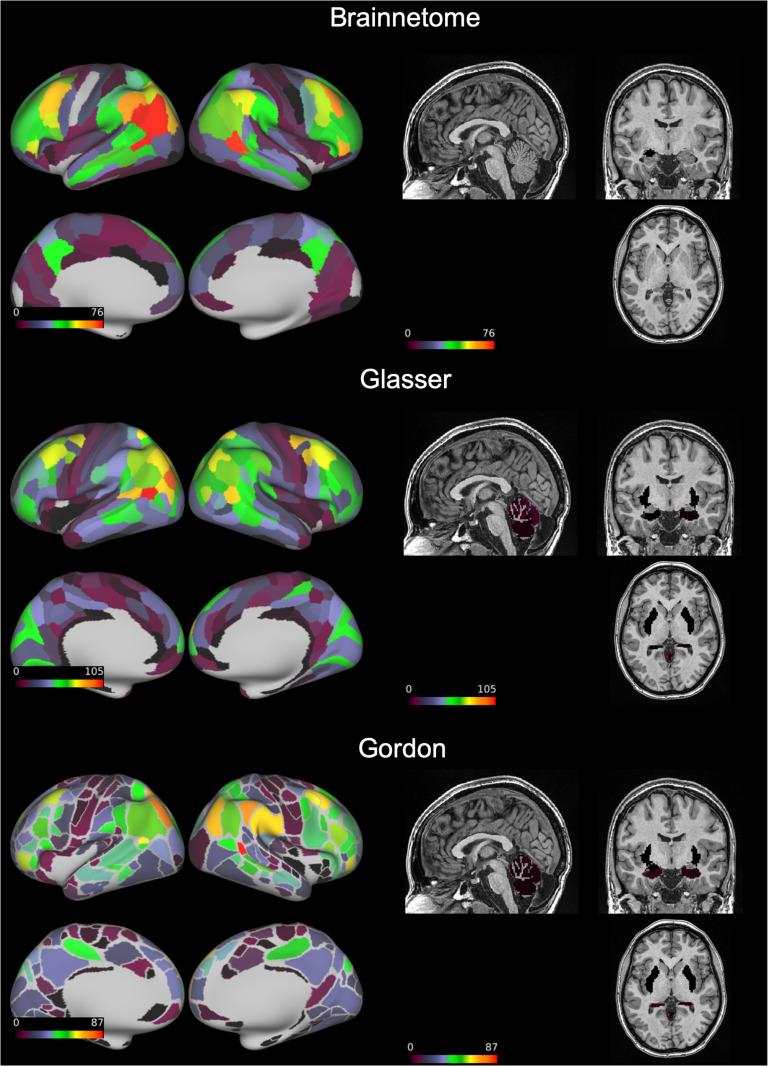
Number of functional connections with “good” reliability by node. On an inflated brain we show, for each node of the Brainnetome, Glasser, and Gordon atlases, the number of functional connections involving the node with intraclass correlation coefficient = 0.60–0.75.

**Figure 4.**
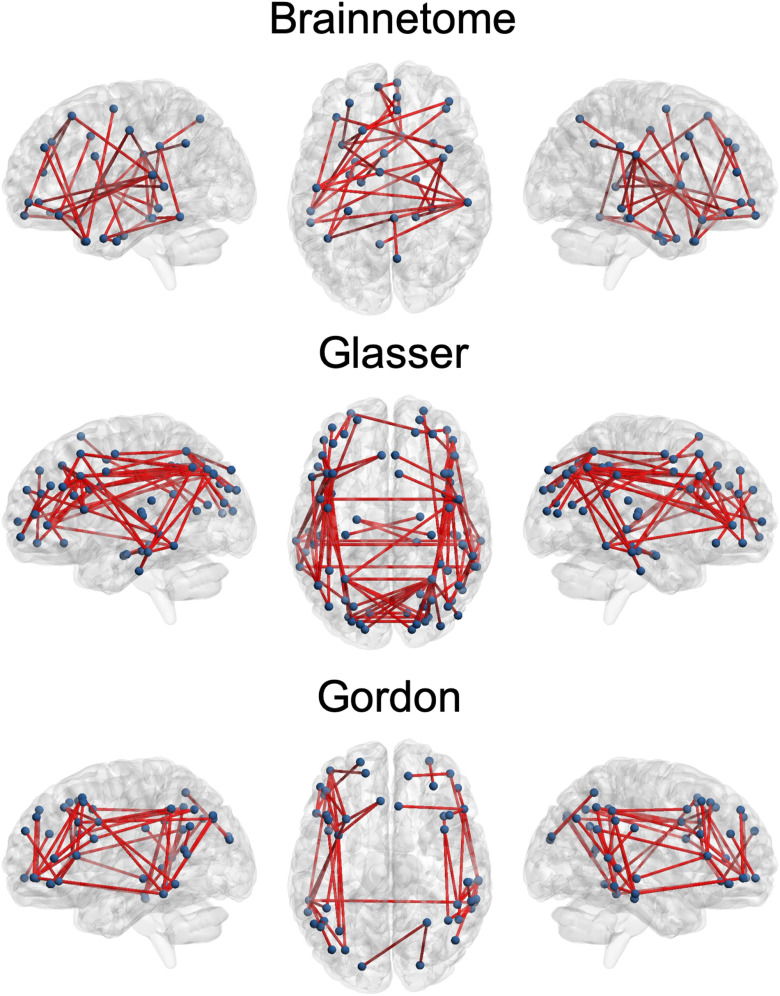
Connections with “excellent” reliability. On a transparent brain we show the connections having “excellent” reliability (intraclass correlation coefficient > 0.75) in the Brainnetome, Glasser, and Gordon atlases. These consistently involved the superior parietal and middle temporal lobes as well as the dorsolateral prefrontal cortex. ICC = intraclass correlation coefficient.

“Good” edges always represented a relatively small portion compared to the total (6–8%) but still numbered in the thousands. These connections mostly involved the inferior parietal and middle temporal lobes, but were also present in the frontal, superior parietal, and occipital lobes (Figure 3). “Excellent” edges were relatively rare and consistently less than a hundred (0.08–0.14% of total connections). These were mostly intrahemispherical and predominantly connected the prefrontal, parietal, and temporal lobes (Figure 4).

### Test-Retest Reliability of Resting-State Networks

**Figure 5.**
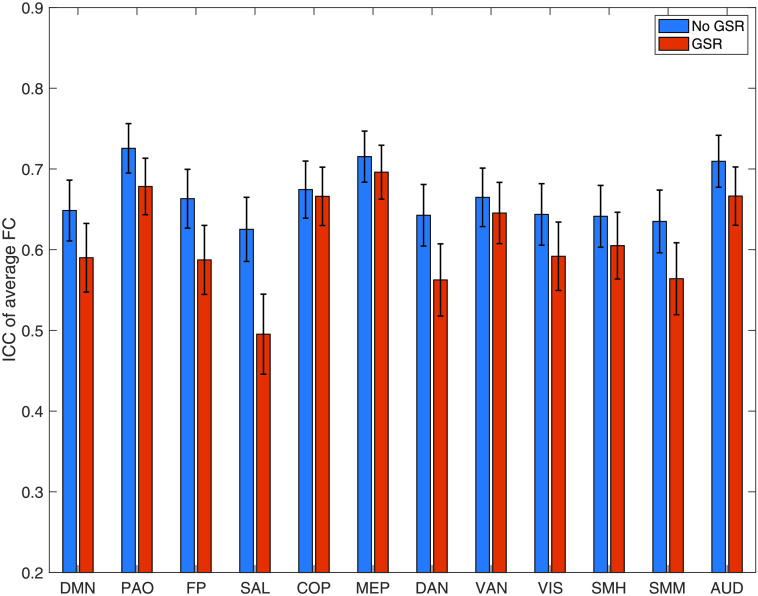
Reliability of known resting-state networks. We show the ICC and confidence intervals for the average connectivity within known resting-state networks defined in accordance to Gordon et al. (2016). ICC = intraclass correlation coefficient; No GSR = no global signal regression; GSR = global signal regression; CI = confidence interval; DMN = default mode network; PAO = parieto-occipital; FP = fronto-parietal; SAL = salience; COP = cingulo-opercular; MEP = medial-parietal; DAN = dorsal attention network; VAN = ventral attention network; VIS = visual; SMH = supplementary motor (hand); SMM = supplementary motor (mouth); AUD = auditory.

Average FC within established resting-state networks always had “good” reliability. The most reliable network was the parieto-occipital (ICC = 0.73), followed by the medial-parietal (ICC = 0.71), and auditory (ICC = 0.71). The least reliable were the salience (ICC = 0.63), dorsal attention (ICC = 0.64), and supplementary motor (mouth) (ICC = 0.64) (Figure 5 and Table 2).

**Table 2.** Reliability of known resting-state networks.

	**ICC**		**Upper CI**		**Lower CI**	
	GSR−	GSR+	GSR−	GSR+	GSR−	GSR+
**DMN**	0.6485	0.5900	0.6862	0.6326	0.6074	0.5439
**PAO**	0.7256	0.6783	0.7562	0.7133	0.6918	0.6398
**FP**	0.6631	0.5873	0.6995	0.6301	0.6233	0.5410
**SAL**	0.6252	0.4953	0.6648	0.5449	0.5820	0.4423
**COP**	0.6744	0.6661	0.7098	0.7022	0.6356	0.6265
**MEP**	0.7153	0.6960	0.7469	0.7294	0.6804	0.6593
**DAN**	0.6426	0.5625	0.6808	0.6072	0.6010	0.5142
**VAN**	0.6648	0.6454	0.7011	0.6834	0.6251	0.6040
**VIS**	0.6437	0.5918	0.6818	0.6342	0.6022	0.5458
**SMH**	0.6413	0.6050	0.6796	0.6464	0.5996	0.5601
**SMM**	0.6350	0.5640	0.6739	0.6086	0.5927	0.5159
**AUD**	0.7095	0.6664	0.7417	0.7025	0.6741	0.6269

*Note*. We show the ICC and confidence intervals for the average connectivity within known resting-state networks defined in accordance to Gordon et al. (2016). ICC = intraclass correlation coefficient; GSR− = no global signal regression; GSR+ = global signal regression; CI = confidence interval; DMN = default mode network; PAO = parieto-occipital; FP = fronto-parietal; SAL= salience; COP = cingulo-opercular; MEP = medial-parietal; DAN = dorsal attention network; VAN = ventral attention network; VIS = visual; SMH = supplementary motor (hand); SMM = supplementary motor (mouth); AUD = auditory.

### Effects of Atlas on Reliability

ICC differed significantly between atlases (Kruskal–Wallis chi-square = 4.96 × 10^3^, *p* < 1 × 10^−32^). The Brainnetome and Glasser atlases had comparable median ICCs (0.47 and 0.46, respectively; Table 1). The proportion of “good” and “excellent” edges was also comparable (8% and 0.14% for both atlases, respectively). However, since the Glasser Atlas has more nodes than the Brainnetome (229 vs. 379), it generated a much larger number of edges (71,631 vs. 26,106). Therefore, using the Glasser Atlas returned a higher absolute number of “good” and “excellent” edges (Figure 4). Median ICC for the Gordon Atlas (352 nodes) was lower (0.41), as well as the proportion of “good” (5%) and “excellent” (0.08%) edges.

### Effect of Global Signal Regression on Reliability

GSR significantly decreased ICC for all atlases (respectively, *Z* = 92.35, *p* < 1 × 10^−32^; *Z* = 188.58, *p* < 1 × 10^−32^; *Z* = 152.21, *p* < 1 × 10^−32^). In particular, in all atlases performing GSR led the reliability of most edges to go from “fair” to “poor”. The ratio of “poor” edges increased from 29% to 53% in the Brainnetome Atlas, 31% to 61% in the Glasser, and from 47% to 69% in the Gordon. Conversely, the ratio of “fair” edges decreased from 63% to 33%, from 61% to 31% and from 47% to 24%, respectively. The ratio of “good” edges, however, marginally increased for the Brainnetome and Gordon atlases (from 8% to 12% and from 6% to 7%, respectively). Also, GSR increased the ratio of “excellent” edges in all atlases: from 0.1% to 2% in the Brainnetome, from 0.1% to 0.4% in the Glasser, and from 0.08% to 0.4% in the Gordon (Table 1 and Figure 1). GSR also reduced the reliability of average FC of resting-state networks (*Z* = 78, *p* = 4.88 × 10^−4^; Table 2 and Figure 5).

### Effect of Edge Strength on Reliability

**Figure 6.**
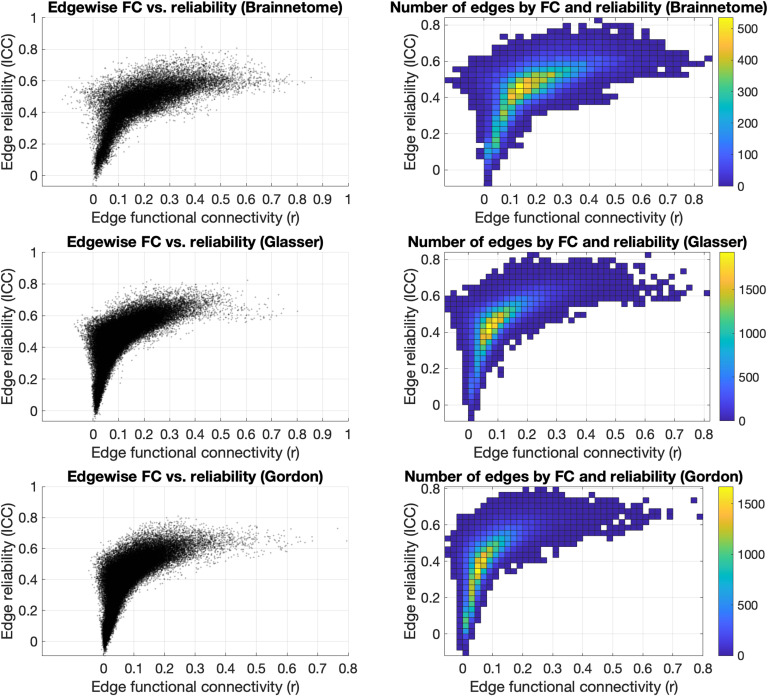
Effects of edge strength on reliability. Plotting the strength of each edge of the Brainnetome, Glasser, and Gordon atlases versus their ICC (left) showed that edges with higher FC were mostly in the “fair” reliability range (ICC = 0.40–0.60). FC of edges with higher reliability (ICC > 0.7) were mostly between *r* = 0.10 and *r* = 0.70, and edges with very low reliability (ICC < 0.30) also had low FC (*r* < 0.20). The distribution of edges (right) had its highest density in an interval of *r* = 0.10–0.30 and a corresponding reliability ranging from “poor” to “fair” (ICC = 0.30–0.50). FC = functional connectivity; ICC = intraclass correlation coefficient; *r* = Pearson correlation coefficient.

Overall, the distribution of edges in the functional connectome had its highest density in an interval of *r* = 0.10–0.30 and a corresponding reliability ranging from “poor” to “fair” (ICC = 0.30–0.50) (Figure 6).

Edge strength was significantly correlated to ICC in all three atlases (rho = 0.76, *p* < 1 × 10^−32^; rho = 0.79, *p* < 1 × 10^−32^; rho = 0.81, *p* < 1 × 10^−32^). However, the FC of edges with “excellent” reliability varied across a wide range of strengths for each of the atlases: Brainnetome, *r* = 0.25–0.61 (median = 0.40), Glasser *r* = 0.12–0.61 (median = 0.34), and Gordon *r* = 0.13–0.44 (median = 0.22). The range was even broader for edges with “good” reliability (respectively, *r* = −0.06–0.85 (median = 0.34), *r* = −0.04–0.80 (median = 0.25), and *r* = −0.01–0.80 (median = 0.21)) as well as “fair” reliability (respectively, *r* = −0.13–0.83 (median = 0.20), *r* = −0.08–0.72 (median = 0.10), and *r* = −0.05–0.60 (median = 0.10)). Edges with poor reliability (ICC < 0.40) tended to have lower FC in all three atlases: respectively, *r* = −0.04–0.45, median = 0.08; *r* = −0.04–0.23, median = 0.04; *r* = −0.03–0.22, median = 0.03.

### Effects of Thresholding on Reliability

**Figure 7.**
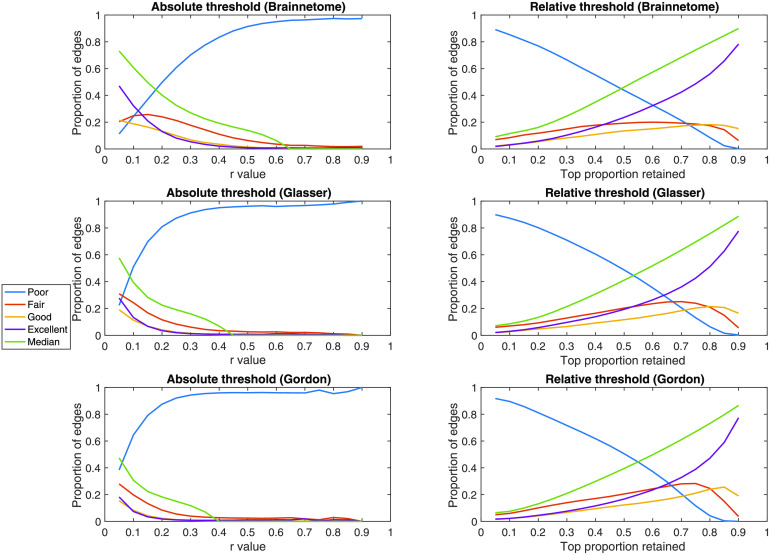
Effects of thresholding on edge retention. In the Brainnetome, Glasser, and Gordon atlases, for each absolute and relative threshold we show the proportion of edges that are consistently retained. As a measure of consistency, we use the number of participants in which the edge was retained at both time points divided by the ones in which it was retained at least once. For convenience, we then use the values defined in Cicchetti (1994) to plot the ratio of edges having poor (ratio < 0.40), fair (ratio = 0.40–0.60), good (ratio = 0.60–0.75), or excellent (ratio > 0.75) consistency. For absolute thresholds (left) all edges below the value are set to 0; for relative ones (right) only the top percent corresponding to the threshold is retained. *r* = Pearson correlation coefficient.

In all three atlases, the median rate of consistent edges significantly decreased as absolute thresholds became more stringent (rho = −0.97, *p* = 1.46 × 10^−12^; rho = −0.90, *p* = 1.77 × 10^−7^; rho = −0.56, *p* = 0.01). The same was true for relative thresholds (rho = −1, *p* = 5.98 × 10^−6^; rho = −1, *p* = 5.98 × 10^−6^; rho = −1, *p* = 5.98 × 10^−6^). The effect of absolute thresholding was a sharp decline in the median ratio of consistent edges, such that from *r* > 0.45 upwards, edges with “excellent” consistency ratio were only around 1% of those retained at least once. Using a relative threshold, the median ratio of consistent edges showed a linear decline as the thresholding became more stringent, mirrored by a proportional increase in edges demonstrating a “poor” consistency ratio (Supporting Information Tables S1–S3 and Figure 7).

**Figure 8.**
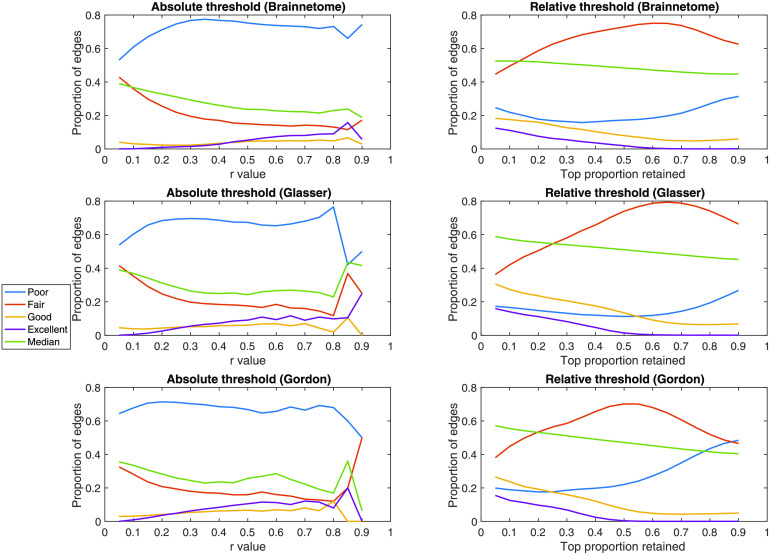
Effects of thresholding on reliability. In the Brainnetome, Glasser, and Gordon atlases, for each absolute and relative threshold we show the proportion of edges having poor (ICC < 0.40), fair (ICC = 0.40–0.60), good (ICC = 0.60–0.75), or excellent (ICC > 0.75) reliability. In this calculation, only subjects for which the edge was retained in both sessions were considered. For absolute thresholds (left) all edges below the value are set to 0; for relative ones (right) only the top percent corresponding to the threshold is retained. ICC = intraclass correlation coefficient; *r* = Pearson correlation coefficient.

For edges retained at both time points, median ICC decreased as a result of increasingly stringent absolute thresholds. This correlation was significant for two out of three atlases (rho = − 0.63, *p* = 0.40 × 10^−2^; rho = −0.18, *p* = 0.48; rho = −0.52, *p* = 0.03). The ICC of most edges that were consistently retained after absolute thresholding was “poor.” With increasingly stringent relative thresholds, median ICC showed a slight increase in all atlases (for the most stringent threshold: 0.06, 0.13, 0.16, respectively) and was significantly correlated to the threshold (rho = 0.95, *p* = 5.95^−6^; rho = 0.99, *p* = 6.27 × 10^−6^; rho = 0.99, *p* = 6.27 × 10^−6^). After relative thresholding, the largest proportion of consistently retained edges was “fairly” reliable. In particular, strict relative thresholds saw a slight increase in consistently retained edges that had “excellent” reliability, mirrored by a decline in “fair” ones (Supporting Information Table S4–S6 and Figure 8).

### Confirmation of Results in Nonrelated Subsample

In a smaller subsample of unrelated participants, without performing GSR, the majority of edges still had “fair” reliability (Supporting Information Table S7 and Figure S3).

When examining resting-state networks, most still had “good” reliability (Supporting Information Table S8 and Figure S4).

The effects of GSR were similar to the ones observed in the full sample: it greatly increased the proportion of “poor” edges, but also caused a small increase in “excellent” ones. For resting-state networks, GSR decreased ICC of most networks (Supporting Information Tables S7–S8 and Figure S4).

The effects of thresholding were also confirmed in the unrelated subsample. We observed a decrease of consistency of edges retained with increasingly strict thresholds (Supporting Information Tables S9–S11 and Figure S6). The median ICC of consistently retained edges was not strongly impacted by thresholding, but strict relative thresholds saw an increase in the proportion of “excellent” edges and a decrease of “fair” ones (Supporting Information Tables S12–S14 and Figure S7).

### Confirmation of Results Using Different ICC Intervals

Using a different binning scheme of ICC with five instead of four classes, our results were substantially unchanged, except for the name of the bin containing most edges (Supporting Information Table S15 and Figure S7). According to this alternative nomenclature, most edges had “moderate” reliability, instead of “fair” for the previous nomenclature. Edges previously classified as having “poor” reliability were better differentiated into a larger proportion with “fair” reliability and a minority with “slight” reliability. Edges with “substantial” and “perfect” reliability were still a minority, and results for them were comparable to the ones for “good” and “excellent” edges in the previous nomenclature.

Consistently with our previous results, average network connectivity had “substantial” reliability.

The effects of GSR were similar to the ones observed with the previous bins: it greatly increased the proportion of “slight” and “fair” edges, but also caused a small increase in “perfect” ones.

The effects of thresholding were also confirmed using a different binning scheme of ICC. We observed a decrease of consistency of edges retained with increasingly strict thresholds (Supporting Information Tables S16–S18 and Figure S8). Strict relative thresholds saw an increase in the proportion of “substantial” edges and a decrease of “moderate” ones (Tables S19–S21 and Figure S9).

## DISCUSSION

In this work, we investigated the short-term test-retest reliability of FC in a large publicly available dataset. We conclude that a small fraction of the intrinsic connectome has good to excellent test-retest reliability. In particular, connectivity between frontal, parietal and temporal areas appears to be especially stable over short timescales. Also, while unreliable edges are generally weak in terms of average FC, the most reliable edges are not necessarily strong. Methodologically, we demonstrate that averaging connections within known networks and not performing global signal regression or thresholding are practices that, in general, improve reliability.

First of all, our results provide theoretical insights about the variability in the functional architecture of the human brain. We show that, regardless of atlas, reliability of the vast majority of edges of the functional connectome is only “fair.” Still, thousands of connections had “good” reliability, and less than a hundred were “excellent.” This is in contrast with a previous study, which reported that the “good” and “excellent” edges combined were more than 25% of the functional connectome when considering 30 minutes of resting state (Elliott et al., 2019). This discrepancy might be due to the much lower sample size of the previous study (*N* = 33), which might not have had sufficient power to detect low ICC values. Indeed, with only two observations per subject, for example, at least 152 subjects are needed to detect an ICC = 0.20 with power = 80% and alpha = 0.05 (Bujang & Baharum, 2017). Importantly, when considering the distribution of the most reliable edges in the brain, some consistent patterns emerged across all atlases. Not surprisingly, connections involving areas prone to susceptibility artifacts, like the subgenual anterior cingulate cortex and inferior temporal lobe, had the lowest average reliability, probably because of lower signal-to-noise ratio. Secondly, in line with recent meta-analytic evidence (Noble et al., 2019), the most reliable connections involved nodes in the superior parietal, middle temporal lobes, and dorsolateral prefrontal cortex. This pattern bears a striking resemblance to what has been labeled the fronto-parietal network (Marek & Dosenbach, 2018). This network is implicated in cognitive control and interacts with other networks implicated in this function, such as the cingulo-opercular and salience networks (Marek et al., 2015; Power et al., 2013). It undergoes functional changes through development, increasing in both strength and flexibility from childhood to adulthood (Chai et al., 2017). Crucially, topography of the fronto-parietal network is highly variable between individuals, which might explain the very high ICC we detected for these connections (Laumann et al., 2015). Interestingly, the average connectivity of the fronto-parietal network, as defined by (Gordon et al., 2016), had only “good” reliability, despite presence of several “excellent” edges in this network as outlined above, suggesting that averaging connections might reduce the impact of the relatively rare “excellent” ones.

Our work informs future studies wanting to focus on portions of the functional connectome that combine high between-subjects reliability with low within-subjects reliability, such as those investigating potential clinical biomarkers or correlations with behavioral measures or self-reports. In particular, the fronto-parietal network has also been shown to be disrupted across a wide range of psychiatric disorders (Cole et al., 2014). This finding, combined with the excellent reliability of FC we have shown for this network, highlights that the functional connections between dorsolateral prefrontal, superior parietal, and middle temporal cortices are excellent potential targets for biomarker development in psychiatry. To aid future studies wishing to focus on specific edges depending on their reliability, we provide connectivity “masks” for each atlas in our GitHub repository, that is, connectivity matrices in which each edge is labeled with its reliability (poor, fair, good, or high), connectivity matrices with the ICC values for each edge, and connectivity matrices with the upper and lower confidence intervals for our ICC estimates (“ICC_matrices/”). Our results also indicate that another viable complimentary approach for biomarker studies is computing average connectivity of known resting-state networks by using an atlas, which consistently returns values that have “good” reliability. This is in line with previous studies which showed, for example, that average connectivity of the default mode network is a good candidate measure for clinical translation and that connectivity within established networks is reliable in general (Ball et al., 2017; Zuo & Xing, 2014). This also offers a very strong reduction of the number of features (from tens of thousands to a dozen), which might be desirable, depending on the study aims.

From a methodological point of view, we were able to test the consequences on reliability of atlas choice, GSR, and thresholding. Concerning the choice of atlas, both the Brainnetome and Glasser atlases had comparable proportion of “good” and “excellent” edges, whereas the Gordon Atlas showed consistently lower reliability. The Glasser Atlas also had the highest absolute number of connections, which led to identification of the highest number of “excellent” edges. Researchers wishing a higher number of reliable edges could consider this atlas, remembering, however, that this comes at the cost of more “poor” and “fair” edges as well. GSR consistently led the majority of edges having “fair” reliability to having “poor” reliability. Also, it consistently reduced ICC of average FC within resting-state networks. This is probably due to the fact that regressing the average signal from each individual subject eliminates the between-subject variability due to differences in the signal baseline, similarly to what has been shown for contrasts between conditions in task fMRI data (Haller et al., 2018). Interestingly, the ratio of “excellent” edges consistently increased, suggesting that some edges might benefit from GSR. Therefore, as previous studies suggested, applying GSR or not can reveal complimentary insights and the choice should be carefully made depending on the study aims (Murphy & Fox, 2017).

Examining the role between FC strength, averaged across sessions, and reliability unveiled a complex relationship. While unreliable edges are generally weak in terms of FC, the opposite is not true: the most reliable edges are not necessarily strong and lie in a broad interval of *r* values (*r* = 0.12–0.61 depending on atlas). Therefore, thresholding the functional connectome based on absolute or relative edge strength had, in general, detrimental consequences on reliability. First of all, applying an increasingly stringent threshold for the two sessions from each subject independently led to an increasingly large amount of connections not being consistently retained. For example, when retaining only connections with *r* > 0.2 or higher, most of the connections that survived thresholding did so in one session but not in the other. The impact of relative thresholding was overall less dramatic compared to absolute thresholding, with the median proportion of reliably kept edges crossing 0.50 only for a threshold of ∼0.50. Examining the impact on ICC of the edges consistently retained after thresholding showed only a slight improvement on the median ICC and ratio of “excellent” edges for increasingly stringent relative thresholds. Also, we calculated ICC only for consistently retained edges, which we showed to be relatively rare. Overall, our results suggest that the practice of thresholding functional connectomes based on edge strength should be avoided in biomarker and correlational studies, especially in the case of absolute thresholds.

Our study is not without limitations. First of all, given that a significant portion of variance in FC is explained by genetics (Adhikari et al., 2018; Elliott et al., 2019; Reineberg et al., 2018), our analyses on the complete Healthy Young Adult dataset might have suffered from reduced between-subjects variability and our results might have overall underestimated reliability. A recent study on the same dataset (Reineberg et al., 2018) concluded that genetic influence on FC is only moderate. In any case, performing the same analyses on the subset of unrelated participants returned results that were overall comparable. Still, the ICC estimates on unrelated participants had broader confidence intervals compared to those of our main analysis because of the lower number of participants. To be certain of removing the effect of family on reliability, a target for future studies is the analysis of very large cohorts of mostly unrelated subjects. Also, the inevitable uncertainty of our ICC estimates should be considered. To allow this, we provide on GitHub for each atlas edge-wise ICC values and their upper and inferior confidence interval bounds. In general, our large sample size made the width of these confidence intervals relatively small and they did not cross the boundary between reliability bins for most edges. Still, we highlight that assignment to a bin of edges with an ICC close to its threshold value is uncertain. In the present work, we also used an ANOVA-derived ICC, which resulted in a very small fraction of results having negative values. Linear-mixed effect implementations of ICC have been recently proposed for functional neuroimaging (Chen et al., 2018). The underlying model being identical, these would have produced identical results on our data, except that the negative values would have been zero. Since our main findings are based on reliability bins and rank statistics, they would be unchanged with these methods. Another limitation is that we assessed reliability over a very short time span. While this allowed us to use a very large sample, our results might not be comparable to the ones from longitudinal designs with repeated measures across weeks or months. Finally, we plan on expanding our analyses to additional parcellations in the future and provide additional connectivity “masks” for atlases that are widely used by the neuroimaging community, such as the Yeo and Desikan-Killiany (Desikan et al., 2006; Thomas Yeo et al., 2011).

## CONCLUSIONS

In conclusion, our findings advance the efforts to identify reliable measures for the field of functional neuroimaging. We demonstrate that a readily identifiable, albeit small, portion of the intrinsic functional connectome is characterized by good to excellent reliability over time. Fronto-temporo-parietal connections appear to be especially reliable over short timescales, and thus suited to delineation of trait-like markers of both normative and disrupted cognitive functions. Averaging of connections within established circuits without thresholding also yields a stable FC metric. Harnessing this knowledge offers one way forward for the study of individual trait-like properties of the human connectome or for disciplines focused on disorders of functional brain connectivity, such as Psychiatry.

## SUPPORTING INFORMATION

Supporting Information for this article is available at https://doi.org/10.1162/netn_a_00148. All data used in this study is in the public domain and was downloaded from https://db.humanconnectome.org/. All code used for the analyses is in the public domain and available at https://github.com/leotozzi88/reliability_study.

## AUTHOR CONTRIBUTIONS

Leonardo Tozzi: Conceptualization; Data curation; Formal analysis; Writing - Original Draft; Writing - Review & Editing. Scott L. Fleming: Conceptualization; Writing - Review & Editing. Zachary D. Taylor: Conceptualization; Writing - Review & Editing. Cooper D. Raterink: Conceptualization; Writing - Review & Editing. Leanne Williams: Conceptualization; Funding acquisition; Project administration; Resources; Supervision; Writing - Original Draft; Writing - Review & Editing.

## FUNDING INFORMATION

Leanne Williams, NIMH, Award ID: U01MH109985.

## TECHNICAL TERMS

Functional connectome: The ensemble of the functional connectivity values between all regions in a brain.
Reliability: We consider a measure “reliable” when it features high between-subjects variability, coupled with low within-subject variability.
Functional connectivity: Pearson correlation coefficient calculated between two blood-oxygen-level dependent (BOLD) timeseries derived from two brain regions.
Functional magnetic resonance imaging (fMRI): A technology that indirectly measures brain activity by detecting changes in the blood-oxygen-level dependent (BOLD) contrast in a magnetic resonance scanner.
Atlas: A set of labels covering the entire brain by which every grayordinate is assigned to a specific brain region.
Global signal regression: A preprocessing practice that involves fitting a linear regression model predicting the timeseries of a region by using as a predictor the average timeseries of all regions. The residuals of this model are then retained for further analysis.
Resting state: A paradigm in which blood-oxygen-level dependent (BOLD) signal is recorded in the absence of any explicit task.
Connectivity matrix: A symmetric matrix with a diagonal of ones representing the functional connectivity values between all brain regions.
Grayordinate: A brain gray matter location that can be represented by a surface vertex (node) or a volume voxel.
