# Supplementary material for: Test-retest reliability of the human functional connectome over consecutive days: identifying highly reliable portions and assessing the impact of methodological choices
Source: Netw Neurosci. 2020 Sep 1;4(3):925–45. doi: 10.1162/netn_a_00148 (PMC7888485; doi:10.1162/netn_a_00148)
Supplement: Supplementary file 1 [file netn-04-925-s001.pdf]

Tozzi, L., Fleming, S. L., Taylor, Z. D., Raterink, C. D., Williams, L. M. (2020). Supporting information for "Test-retest reliability of the human functional connectome over consecutive days: identifying highly reliable portions and assessing the impact of methodological choices." *Network Neuroscience*, *4*(3), 925-945.
https://doi.org/10.1162/netn_a_00148

# Supplementary Material

## Test-retest reliability of the human functional connectome over consecutive days: identifying highly reliable portions and assessing the impact of methodological choices

Leonardo Tozzi[1], Scott L. Fleming[2], Zachary D. Taylor[3], Cooper D. Raterink[3], Leanne M. Williams[1]

[1] Department of Psychiatry and Behavioral Sciences, Stanford University, Stanford, USA
[2] Department of Biomedical Data Science, Stanford University, Stanford, USA
[3] Department of Computer Science, Stanford University, Stanford, USA

## Supplementary Tables

Table S1

| | Absolute | | | | | | | Relative | | | | | | |
|---|---|---|---|---|---|---|---|---|---|---|---|---|---|---|
| **Thres hold** | Median ratio | Min ratio | Max ratio | Poor edges ratio | Fair edges ratio | Good edges ratio | Excellent edges ratio | Median ratio | Min ratio | Max ratio | Poor edges ratio | Fair edges ratio | Good edges ratio | Excellent edges ratio |
| **0.050** | 0.731 | 0.052 | 1.000 | 0.112 | 0.204 | 0.214 | 0.471 | 0.091 | 0.000 | 1.000 | 0.891 | 0.069 | 0.021 | 0.018 |
| **0.100** | 0.607 | 0.000 | 1.000 | 0.242 | 0.248 | 0.188 | 0.322 | 0.115 | 0.000 | 1.000 | 0.853 | 0.084 | 0.033 | 0.030 |
| **0.150** | 0.496 | 0.000 | 1.000 | 0.370 | 0.257 | 0.163 | 0.210 | 0.135 | 0.000 | 1.000 | 0.811 | 0.104 | 0.041 | 0.043 |
| **0.200** | 0.402 | 0.000 | 1.000 | 0.497 | 0.240 | 0.133 | 0.130 | 0.161 | 0.000 | 1.000 | 0.769 | 0.118 | 0.054 | 0.059 |
| **0.250** | 0.325 | 0.000 | 1.000 | 0.607 | 0.212 | 0.099 | 0.081 | 0.200 | 0.000 | 1.000 | 0.719 | 0.133 | 0.070 | 0.078 |
| **0.300** | 0.270 | 0.000 | 1.000 | 0.702 | 0.177 | 0.068 | 0.054 | 0.246 | 0.000 | 1.000 | 0.665 | 0.150 | 0.082 | 0.103 |
| **0.350** | 0.225 | 0.000 | 1.000 | 0.775 | 0.143 | 0.048 | 0.034 | 0.296 | 0.000 | 1.000 | 0.607 | 0.167 | 0.095 | 0.131 |
| **0.400** | 0.192 | 0.000 | 1.000 | 0.834 | 0.110 | 0.035 | 0.021 | 0.350 | 0.000 | 1.000 | 0.552 | 0.177 | 0.108 | 0.163 |
| **0.450** | 0.164 | 0.000 | 1.000 | 0.882 | 0.082 | 0.022 | 0.014 | 0.405 | 0.000 | 1.000 | 0.495 | 0.185 | 0.123 | 0.197 |
| **0.500** | 0.139 | 0.000 | 1.000 | 0.914 | 0.063 | 0.014 | 0.009 | 0.461 | 0.000 | 1.000 | 0.438 | 0.192 | 0.135 | 0.235 |
| **0.550** | 0.108 | 0.000 | 1.000 | 0.936 | 0.048 | 0.009 | 0.007 | 0.519 | 0.000 | 1.000 | 0.383 | 0.198 | 0.142 | 0.277 |
| **0.600** | 0.063 | 0.000 | 1.000 | 0.950 | 0.036 | 0.006 | 0.008 | 0.574 | 0.008 | 1.000 | 0.326 | 0.200 | 0.150 | 0.324 |
| **0.650** | 0.000 | 0.000 | 1.000 | 0.960 | 0.027 | 0.005 | 0.008 | 0.629 | 0.024 | 1.000 | 0.271 | 0.198 | 0.160 | 0.372 |
| **0.700** | 0.000 | 0.000 | 1.000 | 0.963 | 0.027 | 0.003 | 0.007 | 0.684 | 0.029 | 1.000 | 0.212 | 0.193 | 0.171 | 0.424 |
| **0.750** | 0.000 | 0.000 | 1.000 | 0.969 | 0.021 | 0.004 | 0.006 | 0.739 | 0.079 | 1.000 | 0.149 | 0.186 | 0.178 | 0.486 |
| **0.800** | 0.000 | 0.000 | 1.000 | 0.974 | 0.017 | 0.002 | 0.007 | 0.793 | 0.096 | 1.000 | 0.085 | 0.174 | 0.182 | 0.560 |
| **0.850** | 0.000 | 0.000 | 1.000 | 0.971 | 0.017 | 0.002 | 0.010 | 0.846 | 0.169 | 1.000 | 0.024 | 0.143 | 0.175 | 0.657 |
| **0.900** | 0.000 | 0.000 | 1.000 | 0.973 | 0.019 | 0.000 | 0.008 | 0.899 | 0.233 | 1.000 | 0.002 | 0.063 | 0.152 | 0.783 |
| **0.950** | 0.000 | 0.000 | 0.250 | 1.000 | 0.000 | 0.000 | 0.000 | 0.953 | 0.305 | 1.000 | 0.000 | 0.007 | 0.047 | 0.946 |

**Effects of thresholding on edge retention in the Brainnetome atlas.** For each absolute and relative threshold we show the proportion of edges that are consistently retained. As a measure of consistency, we use the number of participants in which the edge was retained at both timepoints divided by the ones in which it was retained at least once. For convenience, we then use the values defined in (1) to compute the ratio of edges having poor (ratio<0.40), fair (ratio=0.40-0.60), good (ratio=0.60-0.75) or excellent (ratio >0.75) consistency. For absolute thresholds, all edges below the value are set to 0, for relative ones (right) only the top percent corresponding to the threshold is retained.

Table S2

| | Absolute | | | | | | | Relative | | | | | | |
|---|---|---|---|---|---|---|---|---|---|---|---|---|---|---|
| **Thres hold** | Median ratio | Min ratio | Max ratio | Poor edges ratio | Fair edges ratio | Good edges ratio | Excellent edges ratio | Median ratio | Min ratio | Max ratio | Poor edges ratio | Fair edges ratio | Good edges ratio | Excellent edges ratio |
| **0.050** | 0.576 | 0.037 | 1.000 | 0.224 | 0.310 | 0.190 | 0.277 | 0.071 | 0.000 | 1.000 | 0.899 | 0.060 | 0.020 | 0.021 |
| **0.100** | 0.393 | 0.000 | 1.000 | 0.511 | 0.243 | 0.113 | 0.132 | 0.086 | 0.000 | 1.000 | 0.873 | 0.070 | 0.028 | 0.030 |
| **0.150** | 0.282 | 0.000 | 1.000 | 0.697 | 0.168 | 0.068 | 0.067 | 0.106 | 0.000 | 1.000 | 0.841 | 0.079 | 0.038 | 0.042 |
| **0.200** | 0.226 | 0.000 | 1.000 | 0.809 | 0.116 | 0.041 | 0.035 | 0.134 | 0.000 | 1.000 | 0.801 | 0.093 | 0.048 | 0.058 |
| **0.250** | 0.191 | 0.000 | 1.000 | 0.873 | 0.083 | 0.024 | 0.020 | 0.171 | 0.000 | 1.000 | 0.756 | 0.111 | 0.056 | 0.077 |
| **0.300** | 0.160 | 0.000 | 1.000 | 0.912 | 0.061 | 0.015 | 0.012 | 0.215 | 0.000 | 1.000 | 0.709 | 0.129 | 0.066 | 0.097 |
| **0.350** | 0.124 | 0.000 | 1.000 | 0.936 | 0.044 | 0.010 | 0.010 | 0.260 | 0.000 | 1.000 | 0.657 | 0.148 | 0.078 | 0.117 |
| **0.400** | 0.069 | 0.000 | 1.000 | 0.950 | 0.035 | 0.006 | 0.009 | 0.308 | 0.000 | 1.000 | 0.605 | 0.165 | 0.092 | 0.139 |
| **0.450** | 0.000 | 0.000 | 1.000 | 0.957 | 0.031 | 0.004 | 0.008 | 0.358 | 0.000 | 1.000 | 0.548 | 0.184 | 0.104 | 0.165 |
| **0.500** | 0.000 | 0.000 | 1.000 | 0.962 | 0.026 | 0.004 | 0.008 | 0.410 | 0.000 | 1.000 | 0.487 | 0.203 | 0.117 | 0.194 |
| **0.550** | 0.000 | 0.000 | 1.000 | 0.965 | 0.025 | 0.003 | 0.007 | 0.464 | 0.000 | 1.000 | 0.423 | 0.220 | 0.130 | 0.227 |
| **0.600** | 0.000 | 0.000 | 1.000 | 0.960 | 0.026 | 0.003 | 0.012 | 0.519 | 0.000 | 1.000 | 0.353 | 0.236 | 0.147 | 0.264 |
| **0.650** | 0.000 | 0.000 | 1.000 | 0.963 | 0.022 | 0.003 | 0.011 | 0.576 | 0.020 | 1.000 | 0.280 | 0.248 | 0.164 | 0.308 |
| **0.700** | 0.000 | 0.000 | 1.000 | 0.967 | 0.022 | 0.003 | 0.008 | 0.635 | 0.028 | 1.000 | 0.205 | 0.251 | 0.184 | 0.360 |
| **0.750** | 0.000 | 0.000 | 1.000 | 0.971 | 0.018 | 0.004 | 0.007 | 0.696 | 0.064 | 1.000 | 0.131 | 0.239 | 0.203 | 0.426 |
| **0.800** | 0.000 | 0.000 | 1.000 | 0.980 | 0.012 | 0.002 | 0.006 | 0.758 | 0.075 | 1.000 | 0.064 | 0.209 | 0.215 | 0.512 |
| **0.850** | 0.000 | 0.000 | 0.501 | 0.991 | 0.009 | 0.000 | 0.000 | 0.822 | 0.132 | 1.000 | 0.016 | 0.149 | 0.207 | 0.628 |
| **0.900** | 0.000 | 0.000 | 0.338 | 1.000 | 0.000 | 0.000 | 0.000 | 0.887 | 0.213 | 1.000 | 0.003 | 0.056 | 0.165 | 0.776 |
| **0.950** | 0.000 | 0.000 | 0.500 | 0.667 | 0.333 | 0.000 | 0.000 | 0.950 | 0.333 | 1.000 | 0.000 | 0.007 | 0.046 | 0.947 |

**Effects of thresholding on edge retention in the Glasser atlas.** For each absolute and relative threshold we show the proportion of edges that are consistently retained. As a measure of consistency, we use the number of participants in which the edge was retained at both timepoints divided by the ones in which it was retained at least once. For convenience, we then use the values defined in (1) to compute the ratio of edges having poor (ratio<0.40), fair (ratio=0.40-0.60), good (ratio=0.60-0.75) or excellent (ratio >0.75) consistency. For absolute thresholds, all edges below the value are set to 0, for relative ones (right) only the top percent corresponding to the threshold is retained.

Table S3

| | Absolute | | | | | | | Relative | | | | | | |
|---|---|---|---|---|---|---|---|---|---|---|---|---|---|---|
| **Threshold** | Median ratio | Min ratio | Max ratio | Poor edges ratio | Fair edges ratio | Good edges ratio | Excellent edges ratio | Median ratio | Min ratio | Max ratio | Poor edges ratio | Fair edges ratio | Good edges ratio | Excellent edges ratio |
| **0.05** | 0.473 | 0.023 | 1.000 | 0.385 | 0.280 | 0.154 | 0.182 | 0.065 | 0.000 | 1.000 | 0.918 | 0.050 | 0.015 | 0.017 |
| **0.10** | 0.307 | 0.000 | 1.000 | 0.646 | 0.198 | 0.083 | 0.074 | 0.075 | 0.000 | 1.000 | 0.895 | 0.060 | 0.023 | 0.022 |
| **0.15** | 0.223 | 0.000 | 1.000 | 0.792 | 0.133 | 0.041 | 0.034 | 0.100 | 0.000 | 1.000 | 0.858 | 0.079 | 0.031 | 0.032 |
| **0.20** | 0.183 | 0.000 | 1.000 | 0.876 | 0.084 | 0.023 | 0.018 | 0.132 | 0.000 | 1.000 | 0.812 | 0.101 | 0.042 | 0.045 |
| **0.25** | 0.151 | 0.000 | 1.000 | 0.921 | 0.055 | 0.012 | 0.011 | 0.168 | 0.000 | 1.000 | 0.765 | 0.121 | 0.054 | 0.059 |
| **0.30** | 0.118 | 0.000 | 1.000 | 0.944 | 0.039 | 0.009 | 0.008 | 0.209 | 0.000 | 1.000 | 0.716 | 0.140 | 0.068 | 0.076 |
| **0.35** | 0.070 | 0.000 | 1.000 | 0.955 | 0.032 | 0.006 | 0.008 | 0.253 | 0.000 | 1.000 | 0.668 | 0.156 | 0.081 | 0.095 |
| **0.40** | 0.000 | 0.000 | 1.000 | 0.960 | 0.028 | 0.004 | 0.008 | 0.299 | 0.000 | 1.000 | 0.618 | 0.171 | 0.096 | 0.116 |
| **0.45** | 0.000 | 0.000 | 1.000 | 0.962 | 0.025 | 0.004 | 0.009 | 0.346 | 0.000 | 1.000 | 0.564 | 0.187 | 0.109 | 0.140 |
| **0.50** | 0.000 | 0.000 | 1.000 | 0.961 | 0.024 | 0.003 | 0.011 | 0.395 | 0.009 | 1.000 | 0.505 | 0.205 | 0.123 | 0.167 |
| **0.55** | 0.000 | 0.000 | 1.000 | 0.962 | 0.023 | 0.004 | 0.011 | 0.446 | 0.037 | 1.000 | 0.442 | 0.223 | 0.135 | 0.200 |
| **0.60** | 0.000 | 0.000 | 1.000 | 0.961 | 0.024 | 0.002 | 0.012 | 0.498 | 0.047 | 1.000 | 0.372 | 0.243 | 0.150 | 0.235 |
| **0.65** | 0.000 | 0.000 | 1.000 | 0.959 | 0.028 | 0.002 | 0.011 | 0.553 | 0.085 | 1.000 | 0.295 | 0.262 | 0.165 | 0.278 |
| **0.70** | 0.000 | 0.000 | 1.000 | 0.960 | 0.019 | 0.003 | 0.018 | 0.610 | 0.108 | 1.000 | 0.205 | 0.281 | 0.186 | 0.328 |
| **0.75** | 0.000 | 0.000 | 1.000 | 0.980 | 0.013 | 0.004 | 0.004 | 0.670 | 0.141 | 1.000 | 0.116 | 0.283 | 0.211 | 0.390 |
| **0.80** | 0.000 | 0.000 | 1.000 | 0.954 | 0.029 | 0.004 | 0.013 | 0.731 | 0.179 | 1.000 | 0.042 | 0.246 | 0.240 | 0.472 |
| **0.85** | 0.000 | 0.000 | 1.000 | 0.968 | 0.021 | 0.000 | 0.011 | 0.797 | 0.240 | 1.000 | 0.004 | 0.150 | 0.256 | 0.590 |
| **0.90** | 0.000 | 0.000 | 0.356 | 1.000 | 0.000 | 0.000 | 0.000 | 0.866 | 0.324 | 1.000 | 0.000 | 0.036 | 0.190 | 0.773 |
| **0.95** | 1.000 | 1.000 | 1.000 | 0.000 | 0.000 | 0.000 | 1.000 | 0.937 | 0.440 | 1.000 | 0.000 | 0.002 | 0.035 | 0.963 |

**Effects of thresholding on edge retention in the Gordon atlas.** For each absolute and relative threshold we show the proportion of edges that are consistently retained. As a measure of consistency, we use the number of participants in which the edge was retained at both timepoints divided by the ones in which it was retained at least once. For convenience, we then use the values defined in (1) to compute the ratio of edges having poor (ratio<0.40), fair (ratio=0.40-0.60), good (ratio=0.60-0.75) or excellent (ratio >0.75) consistency. For absolute thresholds, all edges below the value are set to 0, for relative ones (right) only the top percent corresponding to the threshold is retained.

Table S4

| | Absolute | | | | | | | Relative | | | | | | |
|---|---|---|---|---|---|---|---|---|---|---|---|---|---|---|
| **Threshold** | Median ICC | Min ICC | Max ICC | Poor edges ratio | Fair edges ratio | Good edges ratio | Excellent edges ratio | Median ICC | Min ICC | Max ICC | Poor edges ratio | Fair edges ratio | Good edges ratio | Excellent edges ratio |
| **0.050** | 0.391 | -0.320 | 0.808 | 0.531 | 0.429 | 0.040 | 0.000 | 0.525 | -1.000 | 1.000 | 0.247 | 0.447 | 0.182 | 0.124 |
| **0.100** | 0.367 | -0.956 | 0.997 | 0.609 | 0.359 | 0.031 | 0.001 | 0.525 | -1.000 | 1.000 | 0.219 | 0.495 | 0.176 | 0.111 |
| **0.150** | 0.346 | -1.000 | 0.999 | 0.669 | 0.299 | 0.027 | 0.005 | 0.523 | -1.000 | 1.000 | 0.199 | 0.540 | 0.167 | 0.094 |
| **0.200** | 0.328 | -1.000 | 1.000 | 0.712 | 0.256 | 0.023 | 0.009 | 0.520 | -1.000 | 1.000 | 0.178 | 0.587 | 0.160 | 0.076 |
| **0.250** | 0.311 | -1.000 | 1.000 | 0.747 | 0.219 | 0.022 | 0.012 | 0.514 | -1.000 | 1.000 | 0.169 | 0.626 | 0.143 | 0.063 |
| **0.300** | 0.293 | -1.000 | 1.000 | 0.767 | 0.195 | 0.023 | 0.015 | 0.508 | -1.000 | 1.000 | 0.163 | 0.655 | 0.127 | 0.055 |
| **0.350** | 0.275 | -1.000 | 1.000 | 0.774 | 0.178 | 0.027 | 0.020 | 0.503 | -1.000 | 1.000 | 0.158 | 0.681 | 0.117 | 0.044 |
| **0.400** | 0.260 | -1.000 | 1.000 | 0.768 | 0.170 | 0.034 | 0.028 | 0.497 | -1.000 | 1.000 | 0.162 | 0.699 | 0.103 | 0.036 |
| **0.450** | 0.247 | -1.000 | 1.000 | 0.764 | 0.155 | 0.039 | 0.043 | 0.490 | -1.000 | 1.000 | 0.168 | 0.714 | 0.090 | 0.027 |
| **0.500** | 0.236 | -1.000 | 1.000 | 0.752 | 0.150 | 0.045 | 0.052 | 0.484 | -1.000 | 1.000 | 0.172 | 0.729 | 0.080 | 0.019 |
| **0.550** | 0.235 | -1.000 | 1.000 | 0.743 | 0.145 | 0.048 | 0.064 | 0.478 | -1.000 | 0.997 | 0.176 | 0.744 | 0.070 | 0.010 |
| **0.600** | 0.228 | -1.000 | 1.000 | 0.738 | 0.142 | 0.047 | 0.074 | 0.471 | -0.931 | 0.999 | 0.184 | 0.751 | 0.060 | 0.004 |
| **0.650** | 0.224 | -1.000 | 1.000 | 0.734 | 0.137 | 0.050 | 0.080 | 0.465 | -0.433 | 0.970 | 0.197 | 0.750 | 0.051 | 0.002 |
| **0.700** | 0.222 | -1.000 | 1.000 | 0.730 | 0.142 | 0.047 | 0.080 | 0.459 | -0.269 | 0.827 | 0.214 | 0.737 | 0.049 | 0.001 |
| **0.750** | 0.214 | -1.000 | 1.000 | 0.720 | 0.139 | 0.053 | 0.088 | 0.454 | -0.155 | 0.807 | 0.240 | 0.712 | 0.048 | 0.001 |
| **0.800** | 0.229 | -0.996 | 1.000 | 0.731 | 0.131 | 0.047 | 0.090 | 0.450 | -0.101 | 0.808 | 0.270 | 0.679 | 0.051 | 0.001 |
| **0.850** | 0.239 | -1.000 | 0.994 | 0.661 | 0.115 | 0.067 | 0.158 | 0.447 | -0.048 | 0.808 | 0.297 | 0.648 | 0.054 | 0.001 |
| **0.900** | 0.188 | -0.999 | 0.848 | 0.743 | 0.171 | 0.029 | 0.057 | 0.447 | -0.046 | 0.810 | 0.314 | 0.626 | 0.060 | 0.001 |
| **0.950** | 0.579 | 0.378 | 0.780 | 0.500 | 0.000 | 0.000 | 0.500 | 0.452 | -0.036 | 0.808 | 0.311 | 0.622 | 0.065 | 0.001 |

**Effects of thresholding on ICC in the Brainnetome atlas.** for each absolute and relative threshold we show the proportion of edges having poor (ICC<0.40), fair (ICC=0.40-0.60), good (ICC=0.60-0.75) or excellent (ICC>0.75) reliability. In this calculation, only subjects for which the edge was retained in both sessions were considered. For absolute thresholds (left) all edges below the value are set to 0, for relative ones (right) only the top percent corresponding to the threshold is retained. Abbreviations: ICC=intraclass correlation coefficient.

Table S5

| | Absolute | | | | | | | Relative | | | | | | |
|---|---|---|---|---|---|---|---|---|---|---|---|---|---|---|
| **Threshold** | Median ICC | Min ICC | Max ICC | Poor edges ratio | Fair edges ratio | Good edges ratio | Excellent edges ratio | Median ICC | Min ICC | Max ICC | Poor edges ratio | Fair edges ratio | Good edges ratio | Excellent edges ratio |
| **0.050** | 0.390 | -0.568 | 0.823 | 0.536 | 0.417 | 0.046 | 0.001 | 0.588 | -1.000 | 1.000 | 0.174 | 0.364 | 0.305 | 0.158 |
| **0.100** | 0.369 | -1.000 | 1.000 | 0.601 | 0.355 | 0.039 | 0.005 | 0.573 | -1.000 | 1.000 | 0.167 | 0.422 | 0.272 | 0.140 |
| **0.150** | 0.342 | -1.000 | 1.000 | 0.656 | 0.293 | 0.038 | 0.013 | 0.561 | -1.000 | 1.000 | 0.157 | 0.469 | 0.250 | 0.123 |
| **0.200** | 0.312 | -1.000 | 1.000 | 0.683 | 0.247 | 0.043 | 0.026 | 0.554 | -1.000 | 1.000 | 0.148 | 0.506 | 0.234 | 0.112 |
| **0.250** | 0.287 | -1.000 | 1.000 | 0.692 | 0.219 | 0.048 | 0.041 | 0.547 | -1.000 | 1.000 | 0.140 | 0.545 | 0.219 | 0.097 |
| **0.300** | 0.265 | -1.000 | 1.000 | 0.695 | 0.198 | 0.052 | 0.055 | 0.540 | -1.000 | 1.000 | 0.131 | 0.581 | 0.205 | 0.082 |
| **0.350** | 0.253 | -1.000 | 1.000 | 0.692 | 0.190 | 0.052 | 0.066 | 0.533 | -1.000 | 1.000 | 0.123 | 0.622 | 0.191 | 0.064 |
| **0.400** | 0.251 | -1.000 | 1.000 | 0.684 | 0.185 | 0.058 | 0.073 | 0.526 | -1.000 | 1.000 | 0.120 | 0.657 | 0.176 | 0.047 |
| **0.450** | 0.252 | -1.000 | 1.000 | 0.674 | 0.181 | 0.059 | 0.085 | 0.518 | -0.999 | 1.000 | 0.115 | 0.701 | 0.157 | 0.028 |
| **0.500** | 0.244 | -1.000 | 1.000 | 0.674 | 0.174 | 0.061 | 0.090 | 0.511 | -0.999 | 1.000 | 0.112 | 0.739 | 0.135 | 0.014 |
| **0.550** | 0.255 | -1.000 | 1.000 | 0.658 | 0.166 | 0.067 | 0.109 | 0.503 | -0.992 | 0.998 | 0.113 | 0.768 | 0.112 | 0.007 |
| **0.600** | 0.263 | -1.000 | 1.000 | 0.656 | 0.179 | 0.067 | 0.097 | 0.496 | -0.921 | 0.998 | 0.117 | 0.787 | 0.092 | 0.004 |
| **0.650** | 0.267 | -0.999 | 1.000 | 0.666 | 0.162 | 0.056 | 0.115 | 0.488 | -0.362 | 0.932 | 0.126 | 0.795 | 0.077 | 0.002 |
| **0.700** | 0.271 | -1.000 | 0.994 | 0.680 | 0.168 | 0.063 | 0.090 | 0.480 | -0.190 | 0.869 | 0.141 | 0.790 | 0.068 | 0.001 |
| **0.750** | 0.252 | -0.996 | 0.992 | 0.686 | 0.168 | 0.044 | 0.102 | 0.472 | -0.095 | 0.896 | 0.162 | 0.772 | 0.065 | 0.001 |
| **0.800** | 0.229 | -0.782 | 0.977 | 0.765 | 0.118 | 0.020 | 0.098 | 0.465 | -0.028 | 0.836 | 0.190 | 0.745 | 0.064 | 0.001 |
| **0.850** | 0.434 | -0.970 | 0.868 | 0.421 | 0.368 | 0.105 | 0.105 | 0.458 | -0.067 | 0.823 | 0.226 | 0.707 | 0.066 | 0.001 |
| **0.900** | 0.416 | -0.295 | 0.764 | 0.500 | 0.250 | 0.000 | 0.250 | 0.453 | -0.051 | 0.823 | 0.264 | 0.667 | 0.068 | 0.001 |
| **0.950** | - | - | - | - | - | - | - | 0.452 | -0.034 | 0.824 | 0.295 | 0.632 | 0.071 | 0.001 |

**Effects of thresholding on ICC in the Glasser atlas.** for each absolute and relative threshold we show the proportion of edges having poor (ICC<0.40), fair (ICC=0.40-0.60), good (ICC=0.60-0.75) or excellent (ICC>0.75) reliability. In this calculation, only subjects for which the edge was retained in both sessions were considered. For absolute thresholds (left) all edges below the value are set to 0, for relative ones (right) only the top percent corresponding to the threshold is retained. A dash indicates that ICC could not be computed for that threshold (not enough remaining consistent edges). Abbreviations: ICC=intraclass correlation coefficient.

Table S6

| | Absolute | | | | | | | Relative | | | | | | |
|---|---|---|---|---|---|---|---|---|---|---|---|---|---|---|
| **Threshold** | Median ICC | Min ICC | Max ICC | Poor edges ratio | Fair edges ratio | Good edges ratio | Excellent edges ratio | Median ICC | Min ICC | Max ICC | Poor edges ratio | Fair edges ratio | Good edges ratio | Excellent edges ratio |
| **0.050** | 0.356 | -0.794 | 0.888 | 0.643 | 0.327 | 0.030 | 0.000 | 0.571 | -1.000 | 1.000 | 0.200 | 0.383 | 0.264 | 0.153 |
| **0.100** | 0.335 | -1.000 | 1.000 | 0.678 | 0.282 | 0.031 | 0.009 | 0.553 | -1.000 | 1.000 | 0.190 | 0.450 | 0.235 | 0.125 |
| **0.150** | 0.307 | -1.000 | 1.000 | 0.707 | 0.237 | 0.035 | 0.021 | 0.541 | -1.000 | 1.000 | 0.182 | 0.498 | 0.207 | 0.113 |
| **0.200** | 0.283 | -1.000 | 1.000 | 0.714 | 0.208 | 0.041 | 0.036 | 0.531 | -1.000 | 1.000 | 0.177 | 0.535 | 0.191 | 0.097 |
| **0.250** | 0.260 | -1.000 | 1.000 | 0.712 | 0.192 | 0.047 | 0.049 | 0.522 | -1.000 | 1.000 | 0.176 | 0.563 | 0.175 | 0.086 |
| **0.300** | 0.245 | -1.000 | 1.000 | 0.703 | 0.179 | 0.054 | 0.063 | 0.511 | -1.000 | 1.000 | 0.186 | 0.586 | 0.160 | 0.069 |
| **0.350** | 0.230 | -1.000 | 1.000 | 0.697 | 0.171 | 0.058 | 0.074 | 0.501 | -1.000 | 1.000 | 0.193 | 0.620 | 0.142 | 0.045 |
| **0.400** | 0.236 | -1.000 | 1.000 | 0.685 | 0.169 | 0.062 | 0.085 | 0.491 | -1.000 | 1.000 | 0.198 | 0.656 | 0.121 | 0.025 |
| **0.450** | 0.230 | -1.000 | 1.000 | 0.681 | 0.160 | 0.063 | 0.096 | 0.481 | -0.993 | 1.000 | 0.205 | 0.688 | 0.096 | 0.011 |
| **0.500** | 0.258 | -1.000 | 1.000 | 0.667 | 0.159 | 0.069 | 0.104 | 0.472 | -0.859 | 0.992 | 0.219 | 0.702 | 0.075 | 0.004 |
| **0.550** | 0.270 | -1.000 | 1.000 | 0.644 | 0.178 | 0.064 | 0.114 | 0.463 | -0.482 | 0.962 | 0.239 | 0.703 | 0.057 | 0.001 |
| **0.600** | 0.281 | -1.000 | 1.000 | 0.661 | 0.156 | 0.073 | 0.109 | 0.453 | -0.404 | 0.863 | 0.269 | 0.683 | 0.048 | 0.000 |
| **0.650** | 0.246 | -0.992 | 0.996 | 0.690 | 0.144 | 0.065 | 0.101 | 0.443 | -0.279 | 0.790 | 0.304 | 0.651 | 0.044 | 0.000 |
| **0.700** | 0.221 | -0.997 | 0.999 | 0.663 | 0.134 | 0.081 | 0.122 | 0.434 | -0.205 | 0.791 | 0.347 | 0.610 | 0.043 | 0.000 |
| **0.750** | 0.162 | -1.000 | 0.960 | 0.701 | 0.130 | 0.065 | 0.104 | 0.425 | -0.173 | 0.795 | 0.390 | 0.565 | 0.044 | 0.000 |
| **0.800** | 0.169 | -0.987 | 0.960 | 0.640 | 0.160 | 0.080 | 0.120 | 0.417 | -0.119 | 0.798 | 0.432 | 0.522 | 0.045 | 0.001 |
| **0.850** | 0.249 | 0.123 | 0.475 | 0.750 | 0.250 | 0.000 | 0.000 | 0.410 | -0.122 | 0.801 | 0.463 | 0.489 | 0.047 | 0.001 |
| **0.900** | 0.062 | -0.295 | 0.420 | 0.500 | 0.500 | 0.000 | 0.000 | 0.405 | -0.067 | 0.804 | 0.482 | 0.468 | 0.049 | 0.001 |
| **0.950** | - | - | - | - | - | - | - | 0.404 | -0.062 | 0.806 | 0.487 | 0.461 | 0.051 | 0.001 |

**Effects of thresholding on ICC in the Gordon atlas.** for each absolute and relative threshold we show the proportion of edges having poor (ICC<0.40), fair (ICC=0.40-0.60), good (ICC=0.60-0.75) or excellent (ICC>0.75) reliability. In this calculation, only subjects for which the edge was retained in both sessions were considered. For absolute thresholds (left) all edges below the value are set to 0, for relative ones (right) only the top percent corresponding to the threshold is retained. A dash indicates that ICC could not be computed for that threshold (not enough remaining consistent edges). Abbreviations: ICC=intraclass correlation coefficient.

Table S7

| | | Median ICC | Min ICC | Max ICC | Poor edges ratio | Fair edges ratio | Good edges ratio | Excellent edges ratio |
|---|---|---|---|---|---|---|---|---|
| **BNT** | GSR- | 0.474 | -0.445 | 0.838 | 0.330 | 0.502 | 0.163 | 0.005 |
| | GSR+ | 0.382 | -0.447 | 0.896 | 0.534 | 0.313 | 0.125 | 0.029 |
| **Glasser** | GSR- | 0.457 | -0.297 | 0.870 | 0.355 | 0.511 | 0.131 | 0.003 |
| | GSR+ | 0.344 | -0.297 | 0.900 | 0.610 | 0.297 | 0.085 | 0.009 |
| **Gordon** | GSR- | 0.412 | -0.350 | 0.825 | 0.473 | 0.430 | 0.094 | 0.002 |
| | GSR+ | 0.305 | -0.345 | 0.881 | 0.674 | 0.245 | 0.073 | 0.008 |

**Reliability of edges in the functional connectome in unrelated participants.** We show the median, minimum and maximum ICC of functional connectomes computed using three different atlases, with or without global signal regression. We also show the proportion of edges having poor (ICC<0.40), fair (ICC=0.40-0.60), good (ICC=0.60-0.75) or excellent (ICC>0.75) reliability, defined in accordance to (1). Abbreviations: ICC=intraclass correlation coefficient, GSR-=no global signal regression, GSR+=global signal regression.

Table S8

| | ICC | | Lower CI | | Upper CI | |
|---|---|---|---|---|---|---|
| | GSR- | GSR+ | GSR- | GSR+ | GSR- | GSR+ |
| **DMN** | 0.670 | 0.590 | 0.612 | 0.542 | 0.721 | 0.666 |
| **PAO** | 0.742 | 0.678 | 0.694 | 0.658 | 0.783 | 0.756 |
| **FP** | 0.663 | 0.587 | 0.604 | 0.517 | 0.714 | 0.647 |
| **SAL** | 0.646 | 0.495 | 0.584 | 0.432 | 0.699 | 0.578 |
| **COP** | 0.686 | 0.666 | 0.631 | 0.653 | 0.735 | 0.752 |
| **MEP** | 0.737 | 0.696 | 0.689 | 0.665 | 0.779 | 0.761 |
| **DAN** | 0.645 | 0.562 | 0.584 | 0.521 | 0.699 | 0.650 |
| **VAN** | 0.664 | 0.645 | 0.605 | 0.612 | 0.716 | 0.721 |
| **VIS** | 0.664 | 0.592 | 0.605 | 0.601 | 0.715 | 0.712 |
| **SMH** | 0.674 | 0.605 | 0.617 | 0.564 | 0.725 | 0.683 |
| **SMM** | 0.653 | 0.564 | 0.593 | 0.484 | 0.706 | 0.620 |
| **AUD** | 0.718 | 0.666 | 0.667 | 0.596 | 0.762 | 0.709 |

**Reliability of known resting state networks in unrelated participants.** We show the ICC and confidence intervals for the average connectivity within known resting state networks defined in accordance to (2). Abbreviations: ICC=intraclass correlation coefficient, GSR-=no global signal regression, GSR+=global signal regression, CI=confidence interval, DMN=default mode network, PAO=parieto-occipital, FP=fronto-parietal, SAL=salience, COP=cingulo-opercular, MEP=medial parietal, DAN=dorsal attention network, VAN=ventral attention network, VIS=visual, SMH=supplementary motor (hand), SMM=supplementary motor (mouth), AUD=auditory.

Table S9

| | Absolute | | | | | | | Relative | | | | | | |
|---|---|---|---|---|---|---|---|---|---|---|---|---|---|---|
| **Threshold** | Median ratio | Min ratio | Max ratio | Poor edges ratio | Fair edges ratio | Good edges ratio | Excellent edges ratio | Median ratio | Min ratio | Max ratio | Poor edges ratio | Fair edges ratio | Good edges ratio | Excellent edges ratio |
| **0.050** | 0.735 | 0.028 | 1.000 | 0.110 | 0.199 | 0.213 | 0.477 | 0.111 | 0.000 | 1.000 | 0.863 | 0.088 | 0.025 | 0.023 |
| **0.100** | 0.615 | 0.000 | 1.000 | 0.237 | 0.243 | 0.191 | 0.329 | 0.143 | 0.000 | 1.000 | 0.829 | 0.099 | 0.037 | 0.034 |
| **0.150** | 0.502 | 0.000 | 1.000 | 0.360 | 0.259 | 0.165 | 0.216 | 0.160 | 0.000 | 1.000 | 0.790 | 0.116 | 0.046 | 0.048 |
| **0.200** | 0.410 | 0.000 | 1.000 | 0.485 | 0.242 | 0.136 | 0.137 | 0.182 | 0.000 | 1.000 | 0.754 | 0.125 | 0.058 | 0.063 |
| **0.250** | 0.338 | 0.000 | 1.000 | 0.592 | 0.218 | 0.104 | 0.087 | 0.214 | 0.000 | 1.000 | 0.709 | 0.138 | 0.072 | 0.082 |
| **0.300** | 0.289 | 0.000 | 1.000 | 0.681 | 0.187 | 0.074 | 0.058 | 0.255 | 0.000 | 1.000 | 0.657 | 0.154 | 0.083 | 0.106 |
| **0.350** | 0.250 | 0.000 | 1.000 | 0.754 | 0.156 | 0.052 | 0.037 | 0.303 | 0.000 | 1.000 | 0.601 | 0.168 | 0.097 | 0.134 |
| **0.400** | 0.217 | 0.000 | 1.000 | 0.810 | 0.127 | 0.039 | 0.024 | 0.356 | 0.000 | 1.000 | 0.545 | 0.179 | 0.110 | 0.166 |
| **0.450** | 0.190 | 0.000 | 1.000 | 0.859 | 0.099 | 0.027 | 0.016 | 0.411 | 0.000 | 1.000 | 0.488 | 0.189 | 0.123 | 0.201 |
| **0.500** | 0.167 | 0.000 | 1.000 | 0.895 | 0.077 | 0.017 | 0.012 | 0.466 | 0.000 | 1.000 | 0.431 | 0.195 | 0.135 | 0.239 |
| **0.550** | 0.125 | 0.000 | 1.000 | 0.916 | 0.063 | 0.010 | 0.011 | 0.523 | 0.000 | 1.000 | 0.376 | 0.199 | 0.144 | 0.281 |
| **0.600** | 0.056 | 0.000 | 1.000 | 0.934 | 0.049 | 0.008 | 0.010 | 0.579 | 0.000 | 1.000 | 0.324 | 0.197 | 0.152 | 0.327 |
| **0.650** | 0.000 | 0.000 | 1.000 | 0.945 | 0.039 | 0.007 | 0.010 | 0.634 | 0.000 | 1.000 | 0.267 | 0.197 | 0.160 | 0.375 |
| **0.700** | 0.000 | 0.000 | 1.000 | 0.943 | 0.041 | 0.005 | 0.012 | 0.688 | 0.010 | 1.000 | 0.209 | 0.192 | 0.169 | 0.429 |
| **0.750** | 0.000 | 0.000 | 1.000 | 0.948 | 0.036 | 0.005 | 0.011 | 0.741 | 0.065 | 1.000 | 0.148 | 0.185 | 0.177 | 0.490 |
| **0.800** | 0.000 | 0.000 | 1.000 | 0.953 | 0.036 | 0.002 | 0.009 | 0.795 | 0.080 | 1.000 | 0.085 | 0.171 | 0.180 | 0.564 |
| **0.850** | 0.000 | 0.000 | 1.000 | 0.939 | 0.033 | 0.003 | 0.025 | 0.847 | 0.135 | 1.000 | 0.024 | 0.140 | 0.173 | 0.662 |
| **0.900** | 0.000 | 0.000 | 1.000 | 0.923 | 0.051 | 0.006 | 0.019 | 0.900 | 0.225 | 1.000 | 0.002 | 0.062 | 0.149 | 0.786 |
| **0.950** | 0.000 | 0.000 | 0.444 | 0.889 | 0.111 | 0.000 | 0.000 | 0.954 | 0.325 | 1.000 | 0.000 | 0.007 | 0.048 | 0.946 |

**Effects of thresholding on edge retention in the Brainnetome atlas in unrelated participants.** For each absolute and relative threshold we show the proportion of edges that are consistently retained. As a measure of consistency, we use the number of participants in which the edge was retained at both timepoints divided by the ones in which it was retained at least once. For convenience, we then use the values defined in (1) to compute the ratio of edges having poor (ratio<0.40), fair (ratio=0.40-0.60), good (ratio=0.60-0.75) or excellent (ratio >0.75) consistency. For absolute thresholds, all edges below the value are set to 0, for relative ones (right) only the top percent corresponding to the threshold is retained.

Table S10

| | Absolute | | | | | | | Relative | | | | | | |
|---|---|---|---|---|---|---|---|---|---|---|---|---|---|---|
| **Thres hold** | Median ratio | Min ratio | Max ratio | Poor edges ratio | Fair edges ratio | Good edges ratio | Excellent edges ratio | Median ratio | Min ratio | Max ratio | Poor edges ratio | Fair edges ratio | Good edge s ratio | Excellent edges ratio |
| **0.050** | 0.577 | 0.020 | 1.000 | 0.222 | 0.311 | 0.191 | 0.276 | 0.091 | 0.000 | 1.000 | 0.875 | 0.076 | 0.023 | 0.026 |
| **0.100** | 0.397 | 0.000 | 1.000 | 0.504 | 0.249 | 0.115 | 0.132 | 0.100 | 0.000 | 1.000 | 0.856 | 0.079 | 0.031 | 0.034 |
| **0.150** | 0.292 | 0.000 | 1.000 | 0.689 | 0.175 | 0.069 | 0.067 | 0.120 | 0.000 | 1.000 | 0.830 | 0.086 | 0.040 | 0.044 |
| **0.200** | 0.240 | 0.000 | 1.000 | 0.796 | 0.125 | 0.043 | 0.035 | 0.145 | 0.000 | 1.000 | 0.796 | 0.096 | 0.049 | 0.059 |
| **0.250** | 0.203 | 0.000 | 1.000 | 0.856 | 0.097 | 0.026 | 0.022 | 0.180 | 0.000 | 1.000 | 0.752 | 0.114 | 0.057 | 0.077 |
| **0.300** | 0.167 | 0.000 | 1.000 | 0.891 | 0.077 | 0.016 | 0.016 | 0.220 | 0.000 | 1.000 | 0.705 | 0.132 | 0.065 | 0.098 |
| **0.350** | 0.129 | 0.000 | 1.000 | 0.911 | 0.063 | 0.012 | 0.014 | 0.264 | 0.000 | 1.000 | 0.654 | 0.149 | 0.079 | 0.117 |
| **0.400** | 0.077 | 0.000 | 1.000 | 0.923 | 0.055 | 0.009 | 0.013 | 0.311 | 0.000 | 1.000 | 0.601 | 0.167 | 0.092 | 0.139 |
| **0.450** | 0.000 | 0.000 | 1.000 | 0.929 | 0.050 | 0.007 | 0.014 | 0.361 | 0.000 | 1.000 | 0.545 | 0.184 | 0.105 | 0.165 |
| **0.500** | 0.000 | 0.000 | 1.000 | 0.930 | 0.048 | 0.006 | 0.015 | 0.412 | 0.000 | 1.000 | 0.485 | 0.204 | 0.117 | 0.195 |
| **0.550** | 0.000 | 0.000 | 1.000 | 0.931 | 0.046 | 0.006 | 0.017 | 0.466 | 0.000 | 1.000 | 0.421 | 0.220 | 0.131 | 0.228 |
| **0.600** | 0.000 | 0.000 | 1.000 | 0.926 | 0.047 | 0.006 | 0.020 | 0.521 | 0.000 | 1.000 | 0.352 | 0.236 | 0.147 | 0.265 |
| **0.650** | 0.000 | 0.000 | 1.000 | 0.930 | 0.046 | 0.006 | 0.018 | 0.577 | 0.020 | 1.000 | 0.278 | 0.249 | 0.165 | 0.308 |
| **0.700** | 0.000 | 0.000 | 1.000 | 0.928 | 0.049 | 0.006 | 0.017 | 0.636 | 0.017 | 1.000 | 0.203 | 0.252 | 0.184 | 0.361 |
| **0.750** | 0.000 | 0.000 | 1.000 | 0.902 | 0.066 | 0.005 | 0.027 | 0.696 | 0.080 | 1.000 | 0.130 | 0.239 | 0.204 | 0.427 |
| **0.800** | 0.000 | 0.000 | 1.000 | 0.891 | 0.057 | 0.006 | 0.046 | 0.758 | 0.080 | 1.000 | 0.062 | 0.209 | 0.215 | 0.514 |
| **0.850** | 0.094 | 0.000 | 0.500 | 0.907 | 0.093 | 0.000 | 0.000 | 0.823 | 0.164 | 1.000 | 0.015 | 0.149 | 0.207 | 0.629 |
| **0.900** | 0.000 | 0.000 | 0.347 | 1.000 | 0.000 | 0.000 | 0.000 | 0.887 | 0.218 | 1.000 | 0.002 | 0.055 | 0.166 | 0.777 |
| **0.950** | 0.500 | 0.500 | 0.500 | 0.000 | 1.000 | 0.000 | 0.000 | 0.950 | 0.324 | 1.000 | 0.000 | 0.007 | 0.046 | 0.947 |

**Effects of thresholding on edge retention in the Glasser atlas in unrelated participants.** For each absolute and relative threshold we show the proportion of edges that are consistently retained. As a measure of consistency, we use the number of participants in which the edge was retained at both timepoints divided by the ones in which it was retained at least once. For convenience, we then use the values defined in (1) to compute the ratio of edges having poor (ratio<0.40), fair (ratio=0.40-0.60), good (ratio=0.60-0.75) or excellent (ratio >0.75) consistency. For absolute thresholds, all edges below the value are set to 0, for relative ones (right) only the top percent corresponding to the threshold is retained.

Table S11

| | Absolute | | | | | | | Relative | | | | | | |
|---|---|---|---|---|---|---|---|---|---|---|---|---|---|---|
| **Threshold** | Median ratio | Min ratio | Max ratio | Poor edges ratio | Fair edges ratio | Good edges ratio | Excellent edges ratio | Median ratio | Min ratio | Max ratio | Poor edges ratio | Fair edges ratio | Good edges ratio | Excellent edges ratio |
| **0.050** | 0.474 | 0.000 | 1.000 | 0.382 | 0.281 | 0.156 | 0.181 | 0.077 | 0.000 | 1.000 | 0.899 | 0.062 | 0.018 | 0.021 |
| **0.100** | 0.312 | 0.000 | 1.000 | 0.640 | 0.202 | 0.085 | 0.073 | 0.089 | 0.000 | 1.000 | 0.882 | 0.069 | 0.025 | 0.025 |
| **0.150** | 0.237 | 0.000 | 1.000 | 0.780 | 0.142 | 0.043 | 0.034 | 0.109 | 0.000 | 1.000 | 0.851 | 0.084 | 0.032 | 0.033 |
| **0.200** | 0.200 | 0.000 | 1.000 | 0.860 | 0.097 | 0.024 | 0.019 | 0.140 | 0.000 | 1.000 | 0.808 | 0.104 | 0.043 | 0.045 |
| **0.250** | 0.167 | 0.000 | 1.000 | 0.902 | 0.070 | 0.014 | 0.014 | 0.174 | 0.000 | 1.000 | 0.761 | 0.124 | 0.055 | 0.060 |
| **0.300** | 0.125 | 0.000 | 1.000 | 0.925 | 0.054 | 0.010 | 0.012 | 0.212 | 0.000 | 1.000 | 0.715 | 0.140 | 0.068 | 0.077 |
| **0.350** | 0.059 | 0.000 | 1.000 | 0.932 | 0.047 | 0.008 | 0.013 | 0.256 | 0.000 | 1.000 | 0.666 | 0.157 | 0.082 | 0.096 |
| **0.400** | 0.000 | 0.000 | 1.000 | 0.933 | 0.046 | 0.007 | 0.014 | 0.301 | 0.000 | 1.000 | 0.615 | 0.172 | 0.096 | 0.116 |
| **0.450** | 0.000 | 0.000 | 1.000 | 0.933 | 0.044 | 0.007 | 0.015 | 0.348 | 0.000 | 1.000 | 0.561 | 0.189 | 0.109 | 0.141 |
| **0.500** | 0.000 | 0.000 | 1.000 | 0.929 | 0.045 | 0.005 | 0.021 | 0.397 | 0.000 | 1.000 | 0.504 | 0.205 | 0.123 | 0.168 |
| **0.550** | 0.000 | 0.000 | 1.000 | 0.928 | 0.042 | 0.006 | 0.023 | 0.448 | 0.000 | 1.000 | 0.439 | 0.226 | 0.135 | 0.200 |
| **0.600** | 0.000 | 0.000 | 1.000 | 0.932 | 0.042 | 0.005 | 0.021 | 0.500 | 0.014 | 1.000 | 0.370 | 0.243 | 0.152 | 0.236 |
| **0.650** | 0.000 | 0.000 | 1.000 | 0.924 | 0.048 | 0.006 | 0.022 | 0.554 | 0.071 | 1.000 | 0.292 | 0.262 | 0.167 | 0.279 |
| **0.700** | 0.000 | 0.000 | 1.000 | 0.933 | 0.036 | 0.000 | 0.031 | 0.612 | 0.102 | 1.000 | 0.204 | 0.281 | 0.187 | 0.329 |
| **0.750** | 0.000 | 0.000 | 1.000 | 0.945 | 0.026 | 0.011 | 0.018 | 0.671 | 0.131 | 1.000 | 0.115 | 0.283 | 0.211 | 0.391 |
| **0.800** | 0.000 | 0.000 | 1.000 | 0.876 | 0.079 | 0.011 | 0.034 | 0.733 | 0.190 | 1.000 | 0.041 | 0.245 | 0.240 | 0.474 |
| **0.850** | 0.000 | 0.000 | 1.000 | 0.846 | 0.115 | 0.000 | 0.038 | 0.797 | 0.238 | 1.000 | 0.004 | 0.149 | 0.255 | 0.592 |
| **0.900** | 0.050 | 0.000 | 0.339 | 1.000 | 0.000 | 0.000 | 0.000 | 0.866 | 0.341 | 1.000 | 0.000 | 0.037 | 0.189 | 0.774 |
| **0.950** | 1.000 | 1.000 | 1.000 | 0.000 | 0.000 | 0.000 | 1.000 | 0.937 | 0.447 | 1.000 | 0.000 | 0.002 | 0.036 | 0.962 |

**Effects of thresholding on edge retention in the Gordon atlas in unrelated participants.** For each absolute and relative threshold we show the proportion of edges that are consistently retained. As a measure of consistency, we use the number of participants in which the edge was retained at both timepoints divided by the ones in which it was retained at least once. For convenience, we then use the values defined in (1) to compute the ratio of edges having poor (ratio<0.40), fair (ratio=0.40-0.60), good (ratio=0.60-0.75) or excellent (ratio >0.75) consistency. For absolute thresholds, all edges below the value are set to 0, for relative ones (right) only the top percent corresponding to the threshold is retained. A dash indicates that consistency could not be computed for that threshold (not enough remaining consistent edges).

Table S12

| | Absolute | | | | | | | Relative | | | | | | |
|---|---|---|---|---|---|---|---|---|---|---|---|---|---|---|
| **Threshold** | Median ICC | Min ICC | Max ICC | Poor edges ratio | Fair edges ratio | Good edges ratio | Excellent edges ratio | Median ICC | Min ICC | Max ICC | Poor edges ratio | Fair edges ratio | Good edges ratio | Excellent edges ratio |
| **0.050** | 0.415 | -0.638 | 0.813 | 0.455 | 0.481 | 0.063 | 0.001 | 0.547 | -1.000 | 1.000 | 0.246 | 0.385 | 0.216 | 0.415 |
| **0.100** | 0.396 | -1.000 | 1.000 | 0.513 | 0.433 | 0.051 | 0.002 | 0.547 | -1.000 | 1.000 | 0.225 | 0.414 | 0.230 | 0.396 |
| **0.150** | 0.379 | -1.000 | 1.000 | 0.562 | 0.385 | 0.044 | 0.009 | 0.548 | -1.000 | 1.000 | 0.197 | 0.450 | 0.229 | 0.379 |
| **0.200** | 0.362 | -1.000 | 1.000 | 0.603 | 0.345 | 0.039 | 0.012 | 0.545 | -1.000 | 1.000 | 0.174 | 0.493 | 0.228 | 0.362 |
| **0.250** | 0.344 | -1.000 | 1.000 | 0.644 | 0.303 | 0.036 | 0.017 | 0.540 | -1.000 | 1.000 | 0.167 | 0.534 | 0.213 | 0.344 |
| **0.300** | 0.327 | -1.000 | 1.000 | 0.668 | 0.272 | 0.039 | 0.022 | 0.537 | -1.000 | 1.000 | 0.153 | 0.573 | 0.199 | 0.327 |
| **0.350** | 0.306 | -1.000 | 1.000 | 0.685 | 0.245 | 0.043 | 0.028 | 0.531 | -1.000 | 1.000 | 0.150 | 0.598 | 0.185 | 0.306 |
| **0.400** | 0.290 | -1.000 | 1.000 | 0.690 | 0.222 | 0.047 | 0.041 | 0.526 | -1.000 | 1.000 | 0.150 | 0.622 | 0.172 | 0.290 |
| **0.450** | 0.274 | -1.000 | 1.000 | 0.694 | 0.200 | 0.051 | 0.055 | 0.519 | -1.000 | 1.000 | 0.157 | 0.639 | 0.159 | 0.274 |
| **0.500** | 0.267 | -1.000 | 1.000 | 0.686 | 0.195 | 0.058 | 0.061 | 0.512 | -1.000 | 1.000 | 0.165 | 0.663 | 0.139 | 0.267 |
| **0.550** | 0.263 | -1.000 | 1.000 | 0.675 | 0.188 | 0.057 | 0.081 | 0.505 | -1.000 | 1.000 | 0.168 | 0.684 | 0.127 | 0.263 |
| **0.600** | 0.263 | -1.000 | 1.000 | 0.673 | 0.180 | 0.059 | 0.087 | 0.499 | -0.997 | 1.000 | 0.169 | 0.703 | 0.115 | 0.263 |
| **0.650** | 0.265 | -1.000 | 1.000 | 0.653 | 0.183 | 0.070 | 0.094 | 0.493 | -1.000 | 1.000 | 0.174 | 0.718 | 0.103 | 0.265 |
| **0.700** | 0.256 | -1.000 | 1.000 | 0.679 | 0.159 | 0.064 | 0.098 | 0.486 | -0.882 | 0.910 | 0.181 | 0.727 | 0.090 | 0.256 |
| **0.750** | 0.243 | -0.999 | 1.000 | 0.675 | 0.164 | 0.068 | 0.092 | 0.479 | -0.467 | 0.899 | 0.199 | 0.715 | 0.084 | 0.243 |
| **0.800** | 0.286 | -1.000 | 1.000 | 0.693 | 0.118 | 0.085 | 0.104 | 0.473 | -0.290 | 0.870 | 0.221 | 0.693 | 0.085 | 0.286 |
| **0.850** | 0.331 | -0.967 | 0.994 | 0.624 | 0.128 | 0.094 | 0.154 | 0.468 | -0.145 | 0.813 | 0.247 | 0.663 | 0.088 | 0.331 |
| **0.900** | 0.206 | -0.999 | 0.939 | 0.655 | 0.207 | 0.034 | 0.103 | 0.467 | -0.096 | 0.813 | 0.270 | 0.635 | 0.094 | 0.206 |
| **0.950** | 0.579 | 0.378 | 0.780 | 0.500 | 0.000 | 0.000 | 0.500 | 0.471 | -0.074 | 0.813 | 0.278 | 0.615 | 0.105 | 0.579 |

**Effects of thresholding on ICC in the Brainnetome atlas in unrelated participants.** for each absolute and relative threshold we show the proportion of edges having poor (ICC<0.40), fair (ICC=0.40-0.60), good (ICC=0.60-0.75) or excellent (ICC>0.75) reliability. In this calculation, only subjects for which the edge was retained in both sessions were considered. For absolute thresholds (left) all edges below the value are set to 0, for relative ones (right) only the top percent corresponding to the threshold is retained. Abbreviations: ICC=intraclass correlation coefficient.

Table S13

| | Absolute | | | | | | | Relative | | | | | | |
|---|---|---|---|---|---|---|---|---|---|---|---|---|---|---|
| **Thres hold** | Media n ICC | Min ICC | Max ICC | Poor edges ratio | Fair edges ratio | Good edges ratio | Excellent edges ratio | Media n ICC | Min ICC | Max ICC | Poor edges ratio | Fair edges ratio | Good edges ratio | Excellent edges ratio |
| **0.050** | 0.402 | -0.951 | 0.957 | 0.494 | 0.447 | 0.058 | 0.001 | 0.600 | -1.000 | 1.000 | 0.189 | 0.310 | 0.318 | 0.182 |
| **0.100** | 0.384 | -1.000 | 1.000 | 0.544 | 0.388 | 0.057 | 0.011 | 0.582 | -1.000 | 1.000 | 0.192 | 0.356 | 0.297 | 0.155 |
| **0.150** | 0.362 | -1.000 | 1.000 | 0.582 | 0.330 | 0.065 | 0.024 | 0.571 | -1.000 | 1.000 | 0.189 | 0.393 | 0.277 | 0.141 |
| **0.200** | 0.341 | -1.000 | 1.000 | 0.602 | 0.281 | 0.072 | 0.044 | 0.563 | -1.000 | 1.000 | 0.181 | 0.428 | 0.263 | 0.128 |
| **0.250** | 0.326 | -1.000 | 1.000 | 0.608 | 0.251 | 0.076 | 0.064 | 0.557 | -1.000 | 1.000 | 0.175 | 0.459 | 0.251 | 0.116 |
| **0.300** | 0.307 | -1.000 | 1.000 | 0.618 | 0.232 | 0.074 | 0.077 | 0.550 | -1.000 | 1.000 | 0.167 | 0.491 | 0.241 | 0.101 |
| **0.350** | 0.293 | -1.000 | 1.000 | 0.619 | 0.217 | 0.079 | 0.086 | 0.543 | -1.000 | 1.000 | 0.162 | 0.527 | 0.227 | 0.085 |
| **0.400** | 0.286 | -1.000 | 1.000 | 0.622 | 0.206 | 0.078 | 0.094 | 0.536 | -1.000 | 1.000 | 0.160 | 0.557 | 0.216 | 0.067 |
| **0.450** | 0.293 | -1.000 | 1.000 | 0.611 | 0.202 | 0.084 | 0.104 | 0.529 | -1.000 | 1.000 | 0.155 | 0.596 | 0.200 | 0.049 |
| **0.500** | 0.272 | -1.000 | 1.000 | 0.622 | 0.190 | 0.079 | 0.109 | 0.521 | -1.000 | 1.000 | 0.152 | 0.636 | 0.182 | 0.030 |
| **0.550** | 0.269 | -1.000 | 1.000 | 0.624 | 0.174 | 0.078 | 0.124 | 0.514 | -0.999 | 1.000 | 0.151 | 0.673 | 0.160 | 0.017 |
| **0.600** | 0.291 | -1.000 | 1.000 | 0.607 | 0.193 | 0.083 | 0.116 | 0.507 | -0.993 | 0.992 | 0.151 | 0.703 | 0.137 | 0.009 |
| **0.650** | 0.319 | -0.999 | 1.000 | 0.605 | 0.195 | 0.079 | 0.121 | 0.498 | -0.993 | 0.989 | 0.156 | 0.725 | 0.115 | 0.004 |
| **0.700** | 0.324 | -1.000 | 1.000 | 0.587 | 0.197 | 0.066 | 0.151 | 0.491 | -0.832 | 0.932 | 0.165 | 0.733 | 0.099 | 0.002 |
| **0.750** | 0.310 | -0.999 | 1.000 | 0.650 | 0.190 | 0.030 | 0.130 | 0.483 | -0.281 | 0.921 | 0.180 | 0.730 | 0.089 | 0.002 |
| **0.800** | 0.339 | -0.782 | 0.954 | 0.630 | 0.196 | 0.065 | 0.109 | 0.475 | -0.134 | 0.840 | 0.202 | 0.712 | 0.085 | 0.002 |
| **0.850** | 0.463 | -0.175 | 0.868 | 0.313 | 0.375 | 0.125 | 0.188 | 0.468 | -0.079 | 0.844 | 0.230 | 0.682 | 0.086 | 0.002 |
| **0.900** | 0.506 | 0.349 | 0.808 | 0.333 | 0.333 | 0.000 | 0.333 | 0.463 | -0.107 | 0.834 | 0.259 | 0.652 | 0.087 | 0.002 |
| **0.950** | - | - | - | - | - | - | - | 0.461 | -0.087 | 0.835 | 0.285 | 0.622 | 0.091 | 0.002 |

**Effects of thresholding on ICC in the Glasser atlas in unrelated participants.** for each absolute and relative threshold we show the proportion of edges having poor (ICC<0.40), fair (ICC=0.40-0.60), good (ICC=0.60-0.75) or excellent (ICC>0.75) reliability. In this calculation, only subjects for which the edge was retained in both sessions were considered. For absolute thresholds (left) all edges below the value are set to 0, for relative ones (right) only the top percent corresponding to the threshold is retained. A dash indicates that ICC could not be computed for that threshold (not enough remaining consistent edges). Abbreviations: ICC=intraclass correlation coefficient.

Table S14

| | Absolute | | | | | | | Relative | | | | | | |
|---|---|---|---|---|---|---|---|---|---|---|---|---|---|---|
| **Thres hold** | Median ICC | Min ICC | Max ICC | Poor edges ratio | Fair edges ratio | Good edges ratio | Excellent edges ratio | Median ICC | Min ICC | Max ICC | Poor edges ratio | Fair edges ratio | Good edges ratio | Excellent edges ratio |
| **0.050** | 0.369 | -0.999 | 0.997 | 0.585 | 0.371 | 0.042 | 0.002 | 0.585 | 0.371 | 0.042 | 0.002 | 0.221 | 0.313 | 0.285 |
| **0.100** | 0.352 | -1.000 | 1.000 | 0.610 | 0.322 | 0.050 | 0.017 | 0.610 | 0.322 | 0.050 | 0.017 | 0.219 | 0.374 | 0.259 |
| **0.150** | 0.325 | -1.000 | 1.000 | 0.636 | 0.272 | 0.057 | 0.035 | 0.636 | 0.272 | 0.057 | 0.035 | 0.214 | 0.418 | 0.239 |
| **0.200** | 0.306 | -1.000 | 1.000 | 0.641 | 0.243 | 0.063 | 0.053 | 0.641 | 0.243 | 0.063 | 0.053 | 0.210 | 0.455 | 0.221 |
| **0.250** | 0.286 | -1.000 | 1.000 | 0.644 | 0.219 | 0.069 | 0.068 | 0.644 | 0.219 | 0.069 | 0.068 | 0.212 | 0.485 | 0.201 |
| **0.300** | 0.276 | -1.000 | 1.000 | 0.642 | 0.201 | 0.073 | 0.084 | 0.642 | 0.201 | 0.073 | 0.084 | 0.219 | 0.509 | 0.185 |
| **0.350** | 0.266 | -1.000 | 1.000 | 0.637 | 0.189 | 0.076 | 0.097 | 0.637 | 0.189 | 0.076 | 0.097 | 0.226 | 0.534 | 0.171 |
| **0.400** | 0.271 | -1.000 | 1.000 | 0.628 | 0.187 | 0.075 | 0.110 | 0.628 | 0.187 | 0.075 | 0.110 | 0.233 | 0.563 | 0.156 |
| **0.450** | 0.275 | -1.000 | 1.000 | 0.618 | 0.184 | 0.077 | 0.122 | 0.618 | 0.184 | 0.077 | 0.122 | 0.239 | 0.597 | 0.136 |
| **0.500** | 0.300 | -1.000 | 1.000 | 0.603 | 0.184 | 0.080 | 0.133 | 0.603 | 0.184 | 0.080 | 0.133 | 0.248 | 0.621 | 0.118 |
| **0.550** | 0.288 | -0.999 | 1.000 | 0.614 | 0.186 | 0.092 | 0.109 | 0.614 | 0.186 | 0.092 | 0.109 | 0.261 | 0.638 | 0.096 |
| **0.600** | 0.311 | -1.000 | 0.999 | 0.633 | 0.168 | 0.090 | 0.110 | 0.633 | 0.168 | 0.090 | 0.110 | 0.280 | 0.640 | 0.078 |
| **0.650** | 0.291 | -1.000 | 0.997 | 0.615 | 0.170 | 0.088 | 0.127 | 0.615 | 0.170 | 0.088 | 0.127 | 0.305 | 0.630 | 0.064 |
| **0.700** | 0.309 | -0.997 | 0.999 | 0.606 | 0.139 | 0.088 | 0.168 | 0.606 | 0.139 | 0.088 | 0.168 | 0.339 | 0.603 | 0.057 |
| **0.750** | 0.258 | -1.000 | 0.925 | 0.639 | 0.148 | 0.098 | 0.115 | 0.639 | 0.148 | 0.098 | 0.115 | 0.372 | 0.571 | 0.056 |
| **0.800** | 0.380 | -0.952 | 0.986 | 0.588 | 0.235 | 0.059 | 0.118 | 0.588 | 0.235 | 0.059 | 0.118 | 0.409 | 0.534 | 0.056 |
| **0.850** | 0.133 | -0.577 | 0.752 | 0.600 | 0.200 | 0.000 | 0.200 | 0.600 | 0.200 | 0.000 | 0.200 | 0.440 | 0.501 | 0.057 |
| **0.900** | 0.619 | 0.619 | 0.619 | 0.000 | 0.000 | 1.000 | 0.000 | 0.000 | 0.000 | 1.000 | 0.000 | 0.460 | 0.479 | 0.060 |
| **0.950** | - | - | - | - | - | - | - | - | - | - | - | 0.467 | 0.468 | 0.064 |

**Effects of thresholding on ICC in the Gordon atlas in unrelated participants.** for each absolute and relative threshold we show the proportion of edges having poor (ICC<0.40), fair (ICC=0.40-0.60), good (ICC=0.60-0.75) or excellent (ICC>0.75) reliability. In this calculation, only subjects for which the edge was retained in both sessions were considered. For absolute thresholds (left) all edges below the value are set to 0, for relative ones (right) only the top percent corresponding to the threshold is retained. A dash indicates that ICC could not be computed for that threshold (not enough remaining consistent edges). Abbreviations: ICC=intraclass correlation coefficient.

Table S15

| | | Slight edges ratio | Fair edges ratio | Moderate edges ratio | Substantial edges ratio | Perfect edges ratio |
|---|---|---|---|---|---|---|
| **BNT** | GSR- | 0.076 | 0.212 | 0.627 | 0.084 | 0.000 |
| | GSR+ | 0.201 | 0.328 | 0.333 | 0.134 | 0.004 |
| **Glasser** | GSR- | 0.056 | 0.254 | 0.611 | 0.079 | 0.000 |
| | GSR+ | 0.194 | 0.420 | 0.310 | 0.075 | 0.001 |
| **Gordon** | GSR- | 0.139 | 0.329 | 0.474 | 0.059 | 0.000 |
| | GSR+ | 0.275 | 0.419 | 0.236 | 0.069 | 0.001 |

**Reliability of edges in the functional connectome using different ICC intervals.** We show the proportion of edges having: slight (<0.20), fair (0.20-0.40), moderate (0.40-0.60), substantial (0.60-0.80), and perfect (>0.80) in accordance to (3). Abbreviations: ICC=intraclass correlation coefficient, GSR-=no global signal regression, GSR+=global signal regression.

Table S16

| | Absolute | | | | | Relative | | | | |
|---|---|---|---|---|---|---|---|---|---|---|
| **Threshold** | Slight edges ratio | Fair edges ratio | Moderate edges ratio | Substantial edges ratio | Perfect edges ratio | Slight edges ratio | Fair edges ratio | Moderate edges ratio | Substantial edges ratio | Perfect edges ratio |
| **0.050** | 0.012 | 0.100 | 0.204 | 0.285 | 0.399 | 0.695 | 0.196 | 0.069 | 0.026 | 0.014 |
| **0.100** | 0.076 | 0.166 | 0.248 | 0.252 | 0.258 | 0.651 | 0.202 | 0.084 | 0.040 | 0.023 |
| **0.150** | 0.143 | 0.227 | 0.257 | 0.212 | 0.161 | 0.609 | 0.202 | 0.104 | 0.052 | 0.033 |
| **0.200** | 0.212 | 0.285 | 0.240 | 0.167 | 0.096 | 0.560 | 0.209 | 0.118 | 0.068 | 0.045 |
| **0.250** | 0.283 | 0.324 | 0.212 | 0.121 | 0.060 | 0.499 | 0.219 | 0.133 | 0.087 | 0.061 |
| **0.300** | 0.350 | 0.352 | 0.177 | 0.083 | 0.039 | 0.437 | 0.228 | 0.150 | 0.106 | 0.079 |
| **0.350** | 0.432 | 0.343 | 0.143 | 0.058 | 0.024 | 0.382 | 0.225 | 0.167 | 0.123 | 0.103 |
| **0.400** | 0.518 | 0.316 | 0.110 | 0.041 | 0.015 | 0.329 | 0.223 | 0.177 | 0.142 | 0.129 |
| **0.450** | 0.597 | 0.284 | 0.082 | 0.026 | 0.010 | 0.279 | 0.216 | 0.185 | 0.161 | 0.159 |
| **0.500** | 0.667 | 0.248 | 0.063 | 0.016 | 0.006 | 0.231 | 0.206 | 0.192 | 0.177 | 0.193 |
| **0.550** | 0.727 | 0.209 | 0.048 | 0.011 | 0.005 | 0.184 | 0.199 | 0.198 | 0.192 | 0.228 |
| **0.600** | 0.779 | 0.171 | 0.036 | 0.008 | 0.006 | 0.139 | 0.188 | 0.200 | 0.205 | 0.269 |
| **0.650** | 0.817 | 0.144 | 0.027 | 0.006 | 0.007 | 0.093 | 0.178 | 0.198 | 0.216 | 0.316 |
| **0.700** | 0.836 | 0.127 | 0.027 | 0.004 | 0.006 | 0.045 | 0.167 | 0.193 | 0.229 | 0.366 |
| **0.750** | 0.867 | 0.102 | 0.021 | 0.004 | 0.006 | 0.011 | 0.138 | 0.186 | 0.244 | 0.420 |
| **0.800** | 0.882 | 0.092 | 0.017 | 0.002 | 0.007 | 0.001 | 0.084 | 0.174 | 0.253 | 0.489 |
| **0.850** | 0.880 | 0.091 | 0.017 | 0.002 | 0.010 | 0.000 | 0.024 | 0.143 | 0.255 | 0.578 |
| **0.900** | 0.889 | 0.084 | 0.019 | 0.000 | 0.008 | 0.000 | 0.002 | 0.063 | 0.228 | 0.708 |
| **0.950** | 0.938 | 0.063 | 0.000 | 0.000 | 0.000 | 0.000 | 0.000 | 0.007 | 0.092 | 0.901 |

**Effects of thresholding on edge retention in the Brainnetome atlas using different ICC intervals.** For each absolute and relative threshold we show the proportion of edges that are consistently retained. As a measure of consistency, we use the number of participants in which the edge was retained at both timepoints divided by the ones in which it was retained at least once. For convenience, we then use the values defined in (3) to compute the ratio of edges having slight (<0.20), fair (0.20-0.40), moderate (0.40-0.60), substantial (0.60-0.80), and perfect (>0.80) consistency. For absolute thresholds, all edges below the value are set to 0, for relative ones (right) only the top percent corresponding to the threshold is retained.

Table S17

| | Absolute | | | | | Relative | | | | |
|---|---|---|---|---|---|---|---|---|---|---|
| **Threshold** | Slight edges ratio | Fair edges ratio | Moderate edges ratio | Substantial edges ratio | Perfect edges ratio | Slight edges ratio | Fair edges ratio | Moderate edges ratio | Substantial edges ratio | Perfect edges ratio |
| **0.050** | 0.224 | 0.310 | 0.190 | 0.277 | 0.577 | 0.899 | 0.060 | 0.020 | 0.021 | 0.071 |
| **0.100** | 0.511 | 0.243 | 0.113 | 0.133 | 0.393 | 0.872 | 0.070 | 0.028 | 0.030 | 0.086 |
| **0.150** | 0.697 | 0.168 | 0.068 | 0.067 | 0.282 | 0.841 | 0.079 | 0.038 | 0.042 | 0.106 |
| **0.200** | 0.809 | 0.116 | 0.041 | 0.035 | 0.225 | 0.802 | 0.093 | 0.048 | 0.058 | 0.134 |
| **0.250** | 0.873 | 0.083 | 0.024 | 0.020 | 0.190 | 0.756 | 0.111 | 0.057 | 0.076 | 0.172 |
| **0.300** | 0.912 | 0.061 | 0.015 | 0.012 | 0.160 | 0.709 | 0.129 | 0.066 | 0.097 | 0.215 |
| **0.350** | 0.936 | 0.044 | 0.010 | 0.010 | 0.122 | 0.657 | 0.148 | 0.079 | 0.117 | 0.260 |
| **0.400** | 0.951 | 0.035 | 0.006 | 0.008 | 0.069 | 0.605 | 0.165 | 0.092 | 0.139 | 0.308 |
| **0.450** | 0.957 | 0.031 | 0.004 | 0.008 | 0.000 | 0.548 | 0.184 | 0.104 | 0.165 | 0.358 |
| **0.500** | 0.961 | 0.026 | 0.004 | 0.008 | 0.000 | 0.487 | 0.203 | 0.117 | 0.194 | 0.411 |
| **0.550** | 0.965 | 0.025 | 0.003 | 0.007 | 0.000 | 0.423 | 0.220 | 0.130 | 0.227 | 0.464 |
| **0.600** | 0.959 | 0.026 | 0.003 | 0.012 | 0.000 | 0.354 | 0.236 | 0.147 | 0.264 | 0.519 |
| **0.650** | 0.964 | 0.021 | 0.004 | 0.011 | 0.000 | 0.280 | 0.248 | 0.163 | 0.308 | 0.576 |
| **0.700** | 0.967 | 0.022 | 0.003 | 0.008 | 0.000 | 0.205 | 0.251 | 0.184 | 0.360 | 0.635 |
| **0.750** | 0.971 | 0.018 | 0.004 | 0.006 | 0.000 | 0.131 | 0.239 | 0.203 | 0.426 | 0.696 |
| **0.800** | 0.978 | 0.012 | 0.002 | 0.008 | 0.000 | 0.064 | 0.209 | 0.215 | 0.512 | 0.758 |
| **0.850** | 0.991 | 0.009 | 0.000 | 0.000 | 0.000 | 0.016 | 0.149 | 0.207 | 0.628 | 0.822 |
| **0.900** | 1.000 | 0.000 | 0.000 | 0.000 | 0.000 | 0.003 | 0.056 | 0.165 | 0.777 | 0.887 |
| **0.950** | 0.667 | 0.333 | 0.000 | 0.000 | 0.000 | 0.000 | 0.007 | 0.046 | 0.947 | 0.950 |

**Effects of thresholding on edge retention in the Glasser atlas using different ICC intervals.** For each absolute and relative threshold we show the proportion of edges that are consistently retained. As a measure of consistency, we use the number of participants in which the edge was retained at both timepoints divided by the ones in which it was retained at least once. For convenience, we then use the values defined in (3) to compute the ratio of edges having slight (<0.20), fair (0.20-0.40), moderate (0.40-0.60), substantial (0.60-0.80), and perfect (>0.80) consistency. For absolute thresholds, all edges below the value are set to 0, for relative ones (right) only the top percent corresponding to the threshold is retained.

Table S18

| | Absolute | | | | | Relative | | | | |
|---|---|---|---|---|---|---|---|---|---|---|
| **Threshold** | Slight edges ratio | Fair edges ratio | Moderate edges ratio | Substantial edges ratio | Perfect edges ratio | Slight edges ratio | Fair edges ratio | Moderate edges ratio | Substantial edges ratio | Perfect edges ratio |
| **0.050** | 0.102 | 0.283 | 0.280 | 0.197 | 0.139 | 0.750 | 0.168 | 0.050 | 0.019 | 0.014 |
| **0.100** | 0.288 | 0.357 | 0.198 | 0.102 | 0.055 | 0.732 | 0.163 | 0.060 | 0.028 | 0.017 |
| **0.150** | 0.435 | 0.357 | 0.133 | 0.051 | 0.025 | 0.682 | 0.176 | 0.080 | 0.039 | 0.024 |
| **0.200** | 0.544 | 0.331 | 0.084 | 0.027 | 0.014 | 0.617 | 0.195 | 0.101 | 0.053 | 0.034 |
| **0.250** | 0.630 | 0.291 | 0.055 | 0.015 | 0.009 | 0.552 | 0.213 | 0.121 | 0.068 | 0.046 |
| **0.300** | 0.697 | 0.246 | 0.039 | 0.011 | 0.007 | 0.485 | 0.231 | 0.140 | 0.085 | 0.059 |
| **0.350** | 0.754 | 0.200 | 0.032 | 0.007 | 0.007 | 0.420 | 0.249 | 0.156 | 0.102 | 0.073 |
| **0.400** | 0.791 | 0.169 | 0.028 | 0.005 | 0.008 | 0.352 | 0.266 | 0.171 | 0.121 | 0.091 |
| **0.450** | 0.815 | 0.147 | 0.025 | 0.004 | 0.009 | 0.286 | 0.278 | 0.186 | 0.140 | 0.109 |
| **0.500** | 0.837 | 0.124 | 0.025 | 0.004 | 0.011 | 0.216 | 0.290 | 0.205 | 0.158 | 0.132 |
| **0.550** | 0.853 | 0.109 | 0.023 | 0.004 | 0.010 | 0.146 | 0.296 | 0.224 | 0.177 | 0.157 |
| **0.600** | 0.864 | 0.096 | 0.025 | 0.003 | 0.012 | 0.083 | 0.289 | 0.243 | 0.196 | 0.188 |
| **0.650** | 0.869 | 0.090 | 0.027 | 0.002 | 0.011 | 0.031 | 0.264 | 0.262 | 0.219 | 0.224 |
| **0.700** | 0.876 | 0.083 | 0.019 | 0.002 | 0.019 | 0.004 | 0.201 | 0.281 | 0.245 | 0.269 |
| **0.750** | 0.901 | 0.079 | 0.013 | 0.005 | 0.002 | 0.000 | 0.116 | 0.283 | 0.278 | 0.322 |
| **0.800** | 0.908 | 0.046 | 0.029 | 0.004 | 0.013 | 0.000 | 0.042 | 0.246 | 0.320 | 0.393 |
| **0.850** | 0.937 | 0.032 | 0.021 | 0.000 | 0.011 | 0.000 | 0.004 | 0.149 | 0.352 | 0.494 |
| **0.900** | 0.833 | 0.167 | 0.000 | 0.000 | 0.000 | 0.000 | 0.000 | 0.036 | 0.297 | 0.666 |
| **0.950** | 0.000 | 0.000 | 0.000 | 0.000 | 1.000 | 0.000 | 0.000 | 0.002 | 0.089 | 0.909 |

**Effects of thresholding on edge retention in the Gordon atlas using different ICC intervals.** For each absolute and relative threshold we show the proportion of edges that are consistently retained. As a measure of consistency, we use the number of participants in which the edge was retained at both timepoints divided by the ones in which it was retained at least once. For convenience, we then use the values defined in (3) to compute the ratio of edges having slight (<0.20), fair (0.20-0.40), moderate (0.40-0.60), substantial (0.60-0.80), and perfect (>0.80) consistency. For absolute thresholds, all edges below the value are set to 0, for relative ones (right) only the top percent corresponding to the threshold is retained.

Table S19

| | Absolute | | | | | Relative | | | | |
|---|---|---|---|---|---|---|---|---|---|---|
| **Threshold** | Slight edges ratio | Fair edges ratio | Moderate edges ratio | Substantial edges ratio | Perfect edges ratio | Slight edges ratio | Fair edges ratio | Moderate edges ratio | Substantial edges ratio | Perfect edges ratio |
| **0.050** | 0.099 | 0.432 | 0.429 | 0.040 | 0.000 | 0.114 | 0.133 | 0.447 | 0.212 | 0.095 |
| **0.100** | 0.125 | 0.484 | 0.359 | 0.031 | 0.000 | 0.096 | 0.123 | 0.495 | 0.206 | 0.080 |
| **0.150** | 0.159 | 0.511 | 0.299 | 0.028 | 0.003 | 0.080 | 0.119 | 0.540 | 0.192 | 0.069 |
| **0.200** | 0.199 | 0.512 | 0.256 | 0.025 | 0.008 | 0.068 | 0.109 | 0.587 | 0.182 | 0.054 |
| **0.250** | 0.247 | 0.499 | 0.219 | 0.025 | 0.010 | 0.061 | 0.108 | 0.626 | 0.161 | 0.045 |
| **0.300** | 0.298 | 0.470 | 0.195 | 0.026 | 0.012 | 0.057 | 0.107 | 0.655 | 0.142 | 0.039 |
| **0.350** | 0.346 | 0.429 | 0.178 | 0.032 | 0.015 | 0.054 | 0.104 | 0.681 | 0.129 | 0.032 |
| **0.400** | 0.388 | 0.380 | 0.170 | 0.041 | 0.021 | 0.054 | 0.108 | 0.699 | 0.113 | 0.026 |
| **0.450** | 0.421 | 0.343 | 0.155 | 0.048 | 0.034 | 0.053 | 0.115 | 0.714 | 0.099 | 0.019 |
| **0.500** | 0.448 | 0.304 | 0.150 | 0.057 | 0.041 | 0.050 | 0.123 | 0.729 | 0.086 | 0.013 |
| **0.550** | 0.450 | 0.293 | 0.145 | 0.059 | 0.052 | 0.044 | 0.132 | 0.744 | 0.074 | 0.006 |
| **0.600** | 0.459 | 0.279 | 0.142 | 0.060 | 0.060 | 0.034 | 0.150 | 0.751 | 0.063 | 0.002 |
| **0.650** | 0.471 | 0.263 | 0.137 | 0.062 | 0.067 | 0.028 | 0.169 | 0.750 | 0.052 | 0.001 |
| **0.700** | 0.472 | 0.259 | 0.142 | 0.056 | 0.072 | 0.023 | 0.191 | 0.737 | 0.049 | 0.000 |
| **0.750** | 0.484 | 0.235 | 0.139 | 0.066 | 0.076 | 0.020 | 0.219 | 0.712 | 0.049 | 0.000 |
| **0.800** | 0.467 | 0.264 | 0.131 | 0.074 | 0.063 | 0.020 | 0.250 | 0.679 | 0.051 | 0.000 |
| **0.850** | 0.479 | 0.182 | 0.115 | 0.079 | 0.145 | 0.022 | 0.276 | 0.648 | 0.055 | 0.000 |
| **0.900** | 0.514 | 0.229 | 0.171 | 0.057 | 0.029 | 0.027 | 0.287 | 0.626 | 0.060 | 0.000 |
| **0.950** | 0.000 | 0.500 | 0.000 | 0.500 | 0.000 | 0.048 | 0.263 | 0.622 | 0.066 | 0.000 |

**Effects of thresholding on ICC in the Brainnetome atlas using different ICC intervals.** For each absolute and relative threshold we show the proportion of edges having slight (<0.20), fair (0.20-0.40), moderate (0.40-0.60), substantial (0.60-0.80), and perfect (>0.80) consistency. In this calculation, only subjects for which the edge was retained in both sessions were considered. For absolute thresholds (left) all edges below the value are set to 0, for relative ones (right) only the top percent corresponding to the threshold is retained. Abbreviations: ICC=intraclass correlation coefficient.

Table S20

| | Absolute | | | | | Relative | | | | |
|---|---|---|---|---|---|---|---|---|---|---|
| **Threshold** | Slight edges ratio | Fair edges ratio | Moderate edges ratio | Substantial edges ratio | Perfect edges ratio | Slight edges ratio | Fair edges ratio | Moderate edges ratio | Substantial edges ratio | Perfect edges ratio |
| **0.050** | 0.072 | 0.467 | 0.415 | 0.046 | 0.000 | 0.096 | 0.077 | 0.363 | 0.346 | 0.118 |
| **0.100** | 0.135 | 0.468 | 0.353 | 0.040 | 0.003 | 0.085 | 0.081 | 0.420 | 0.310 | 0.103 |
| **0.150** | 0.232 | 0.426 | 0.292 | 0.042 | 0.010 | 0.077 | 0.080 | 0.468 | 0.283 | 0.091 |
| **0.200** | 0.318 | 0.366 | 0.247 | 0.050 | 0.019 | 0.069 | 0.079 | 0.505 | 0.266 | 0.081 |
| **0.250** | 0.383 | 0.310 | 0.219 | 0.057 | 0.032 | 0.060 | 0.079 | 0.545 | 0.246 | 0.069 |
| **0.300** | 0.421 | 0.275 | 0.197 | 0.063 | 0.043 | 0.052 | 0.080 | 0.581 | 0.231 | 0.056 |
| **0.350** | 0.441 | 0.253 | 0.189 | 0.064 | 0.053 | 0.045 | 0.078 | 0.622 | 0.211 | 0.043 |
| **0.400** | 0.448 | 0.238 | 0.184 | 0.071 | 0.059 | 0.040 | 0.080 | 0.657 | 0.193 | 0.029 |
| **0.450** | 0.449 | 0.225 | 0.181 | 0.074 | 0.070 | 0.033 | 0.082 | 0.701 | 0.168 | 0.016 |
| **0.500** | 0.458 | 0.215 | 0.176 | 0.077 | 0.074 | 0.027 | 0.086 | 0.740 | 0.141 | 0.008 |
| **0.550** | 0.452 | 0.204 | 0.167 | 0.088 | 0.088 | 0.021 | 0.093 | 0.767 | 0.115 | 0.003 |
| **0.600** | 0.442 | 0.212 | 0.184 | 0.084 | 0.079 | 0.017 | 0.101 | 0.787 | 0.093 | 0.001 |
| **0.650** | 0.436 | 0.228 | 0.163 | 0.080 | 0.093 | 0.015 | 0.113 | 0.793 | 0.078 | 0.000 |
| **0.700** | 0.453 | 0.227 | 0.160 | 0.087 | 0.073 | 0.013 | 0.130 | 0.787 | 0.069 | 0.000 |
| **0.750** | 0.420 | 0.283 | 0.145 | 0.058 | 0.094 | 0.014 | 0.151 | 0.770 | 0.066 | 0.000 |
| **0.800** | 0.471 | 0.294 | 0.118 | 0.039 | 0.078 | 0.015 | 0.179 | 0.741 | 0.065 | 0.000 |
| **0.850** | 0.158 | 0.263 | 0.368 | 0.158 | 0.053 | 0.018 | 0.212 | 0.704 | 0.067 | 0.000 |
| **0.900** | 0.250 | 0.250 | 0.250 | 0.250 | 0.000 | 0.024 | 0.244 | 0.663 | 0.069 | 0.000 |
| **0.950** | - | - | - | - | - | 0.038 | 0.260 | 0.630 | 0.073 | 0.000 |

**Effects of thresholding on ICC in the Glasser atlas using different ICC intervals.** For each absolute and relative threshold we show the proportion of edges having slight (<0.20), fair (0.20-0.40), moderate (0.40-0.60), substantial (0.60-0.80), and perfect (>0.80) consistency. In this calculation, only subjects for which the edge was retained in both sessions were considered. For absolute thresholds (left) all edges below the value are set to 0, for relative ones (right) only the top percent corresponding to the threshold is retained. A dash indicates that ICC could not be computed for that threshold (not enough remaining consistent edges). Abbreviations: ICC=intraclass correlation coefficient.

Table S21

| | Absolute | | | | | Relative | | | | |
|---|---|---|---|---|---|---|---|---|---|---|
| **Thresh old** | Slight edges ratio | Fair edges ratio | Moderate edges ratio | Substantial edges ratio | Perfect edges ratio | Slight edges ratio | Fair edges ratio | Moderate edges ratio | Substantial edges ratio | Perfect edges ratio |
| **0.050** | 0.152 | 0.493 | 0.326 | 0.030 | 0.000 | 0.105 | 0.094 | 0.381 | 0.304 | 0.116 |
| **0.100** | 0.236 | 0.442 | 0.282 | 0.033 | 0.007 | 0.091 | 0.098 | 0.448 | 0.269 | 0.093 |
| **0.150** | 0.321 | 0.386 | 0.236 | 0.040 | 0.016 | 0.083 | 0.099 | 0.497 | 0.237 | 0.083 |
| **0.200** | 0.381 | 0.333 | 0.208 | 0.050 | 0.028 | 0.074 | 0.102 | 0.534 | 0.218 | 0.071 |
| **0.250** | 0.424 | 0.287 | 0.194 | 0.056 | 0.039 | 0.072 | 0.104 | 0.564 | 0.199 | 0.061 |
| **0.300** | 0.449 | 0.254 | 0.180 | 0.067 | 0.050 | 0.071 | 0.115 | 0.586 | 0.181 | 0.048 |
| **0.350** | 0.469 | 0.228 | 0.172 | 0.071 | 0.061 | 0.070 | 0.123 | 0.620 | 0.157 | 0.030 |
| **0.400** | 0.462 | 0.223 | 0.168 | 0.080 | 0.067 | 0.061 | 0.137 | 0.656 | 0.131 | 0.015 |
| **0.450** | 0.472 | 0.209 | 0.159 | 0.081 | 0.079 | 0.052 | 0.154 | 0.687 | 0.101 | 0.006 |
| **0.500** | 0.448 | 0.221 | 0.159 | 0.086 | 0.086 | 0.045 | 0.176 | 0.702 | 0.076 | 0.001 |
| **0.550** | 0.433 | 0.214 | 0.176 | 0.087 | 0.090 | 0.041 | 0.200 | 0.701 | 0.058 | 0.000 |
| **0.600** | 0.410 | 0.248 | 0.160 | 0.095 | 0.088 | 0.038 | 0.233 | 0.680 | 0.048 | 0.000 |
| **0.650** | 0.443 | 0.241 | 0.151 | 0.087 | 0.078 | 0.039 | 0.267 | 0.649 | 0.044 | 0.000 |
| **0.700** | 0.468 | 0.197 | 0.133 | 0.104 | 0.098 | 0.042 | 0.308 | 0.607 | 0.043 | 0.000 |
| **0.750** | 0.500 | 0.192 | 0.128 | 0.115 | 0.064 | 0.048 | 0.346 | 0.562 | 0.044 | 0.000 |
| **0.800** | 0.520 | 0.160 | 0.120 | 0.120 | 0.080 | 0.057 | 0.378 | 0.519 | 0.046 | 0.000 |
| **0.850** | 0.400 | 0.200 | 0.200 | 0.200 | 0.000 | 0.073 | 0.393 | 0.486 | 0.048 | 0.000 |
| **0.900** | 0.500 | 0.000 | 0.500 | 0.000 | 0.000 | 0.095 | 0.390 | 0.465 | 0.050 | 0.000 |
| **0.950** | - | - | - | - | - | 0.119 | 0.370 | 0.459 | 0.052 | 0.000 |

**Effects of thresholding on ICC in the Gordon atlas using different ICC intervals.** For each absolute and relative threshold we show the proportion of edges having slight (<0.20), fair (0.20-0.40), moderate (0.40-0.60), substantial (0.60-0.80), and perfect (>0.80) consistency. In this calculation, only subjects for which the edge was retained in both sessions were considered. For absolute thresholds (left) all edges below the value are set to 0, for relative ones (right) only the top percent corresponding to the threshold is retained. A dash indicates that ICC could not be computed for that threshold (not enough remaining consistent edges). Abbreviations: ICC=intraclass correlation coefficient.

# Supplementary Figures

Figure S1

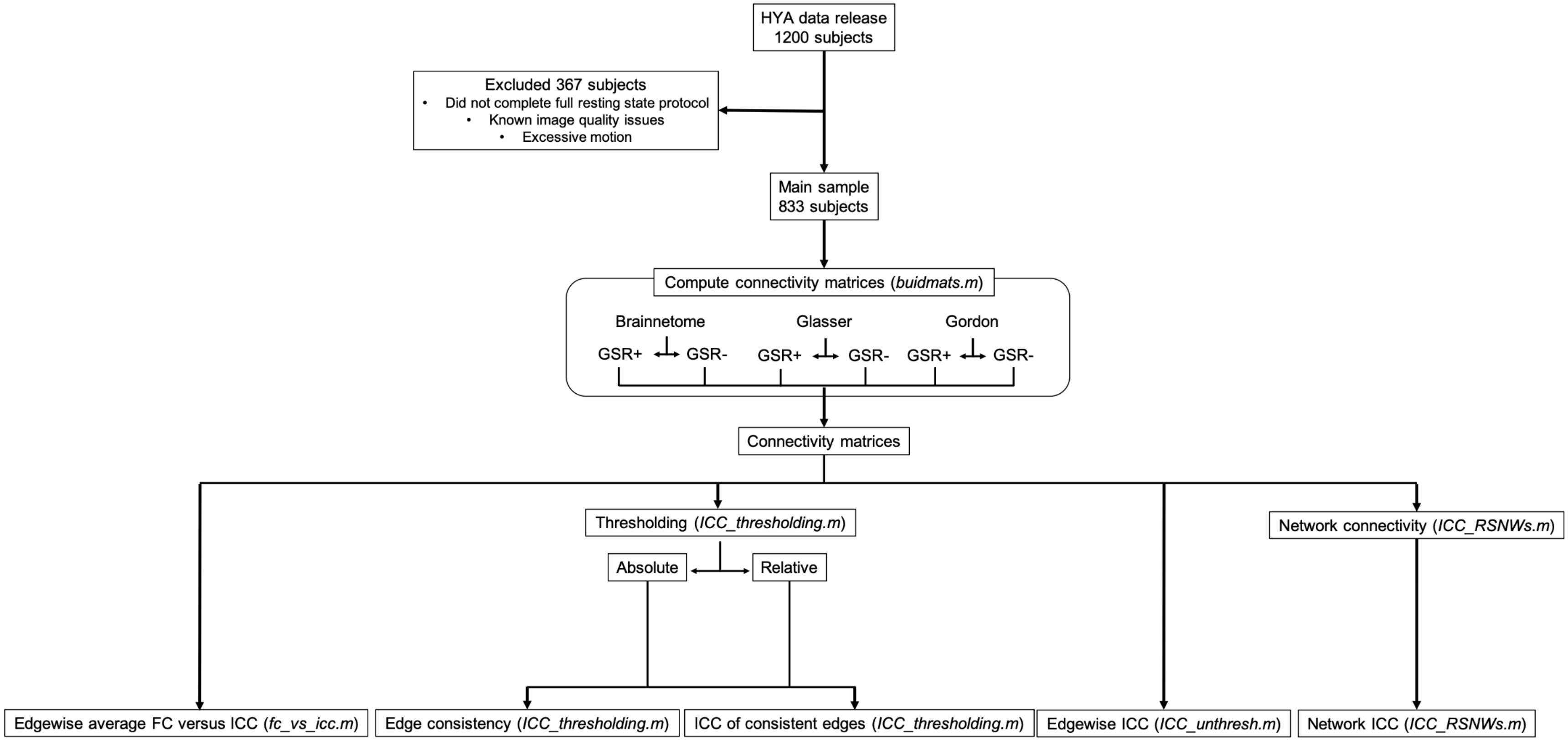


**Study flow.** In this diagram, the selection of the final sample and analysis steps are summarized. For each analysis step, we report the name of the GitHub script available at https://github.com/leotozzi88/repeatability_study to reproduce the results. Abbreviations: HYA=healthy young adult, GSR-=no global signal regression, GSR+=global signal regression, ICC=intraclass correlation coefficient, FC=functional connectivity.

Figure S2

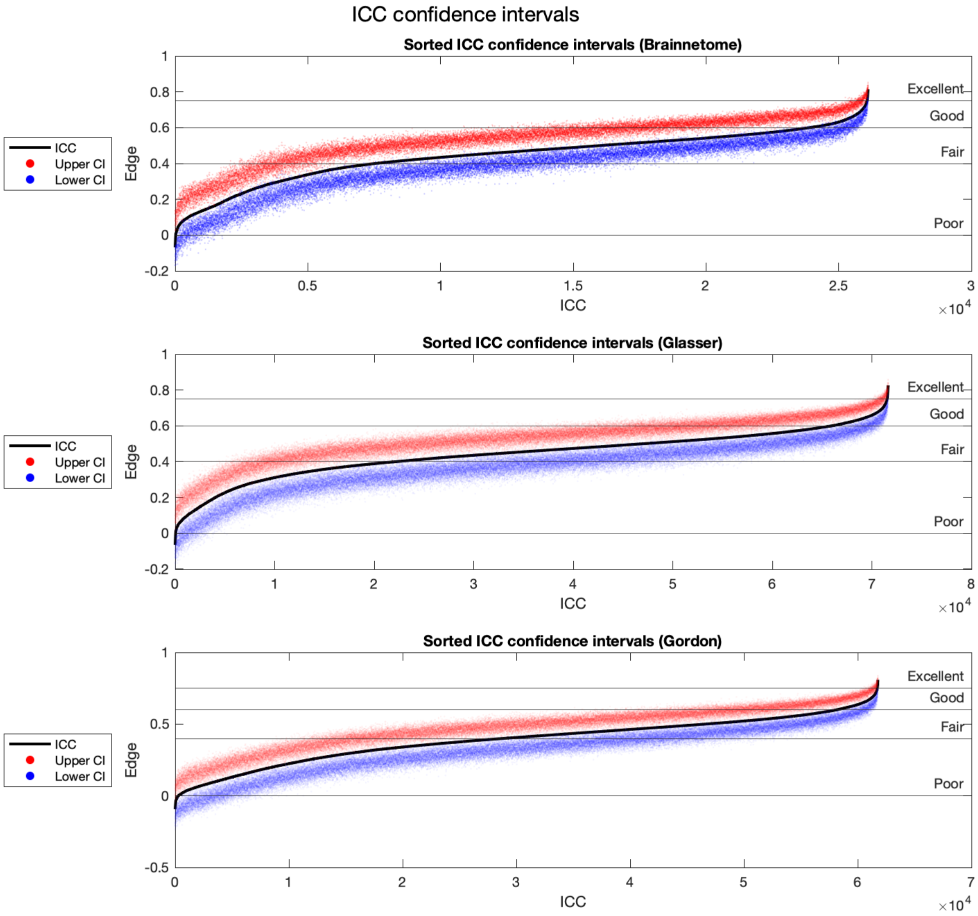


**Confidence intervals of edge-wise ICC.** For the Brainnetome, Glasser and Gordon atlases we show the upper (red) and lower bounds (blue) of the confidence interval of ICC estimates. We also show the boundaries of ICC bins as defined by (1): poor (ICC<0.40), fair (ICC=0.40-0.60), good (ICC=0.60-0.75) or excellent (ICC>0.75). ICC were sorted in increasing order for visualization. Abbreviations: ICC=intraclass correlation coefficient.

Figure S3

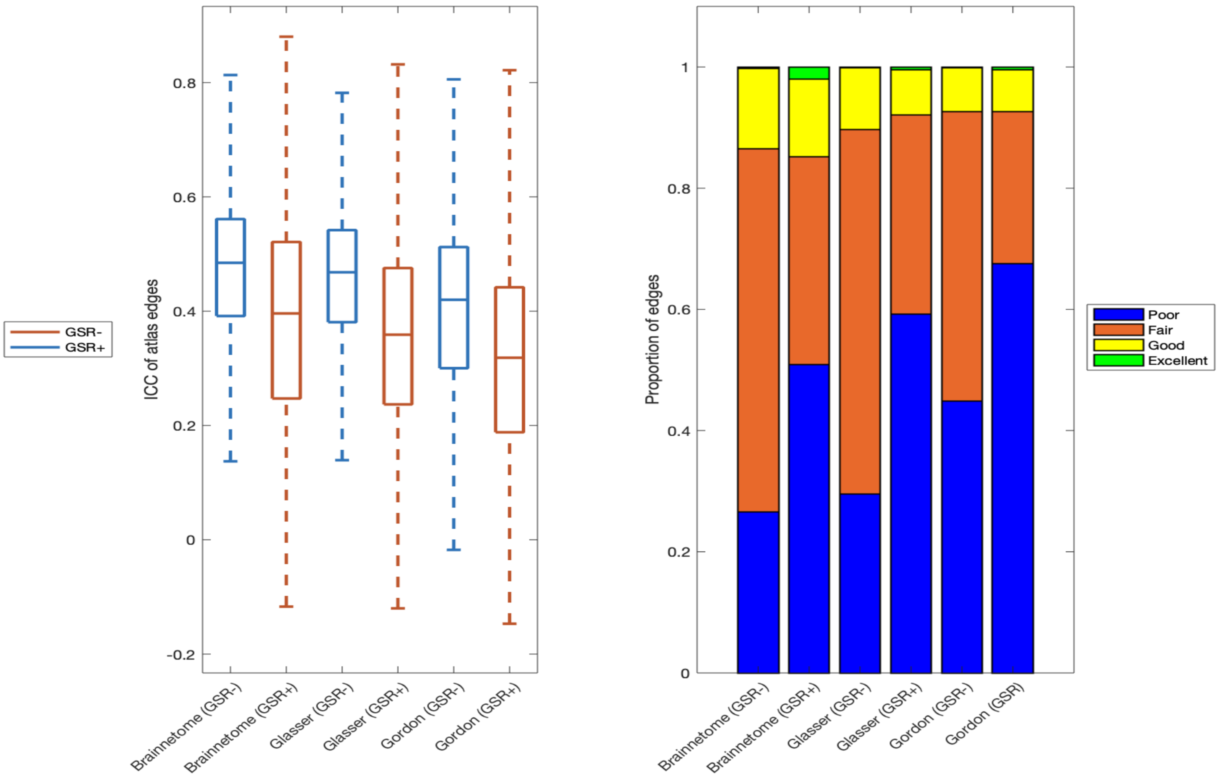


**Reliability of functional connectome edges in unrelated participants.** Left: For the Brainnetome, Glasser and Gordon atlases with and without performing GSR we show boxplots of ICC of all atlas edges. Right: For the Brainnetome, Glasser and Gordon atlases with and without performing GSR we show the proportion of edges having poor (ICC<0.40), fair (ICC=0.40-0.60), good (ICC=0.60-0.75) or excellent (ICC>0.75) reliability, defined in accordance to (1). Abbreviations: ICC=intraclass correlation coefficient, GSR-=no global signal regression, GSR+=global signal regression.

Figure S4

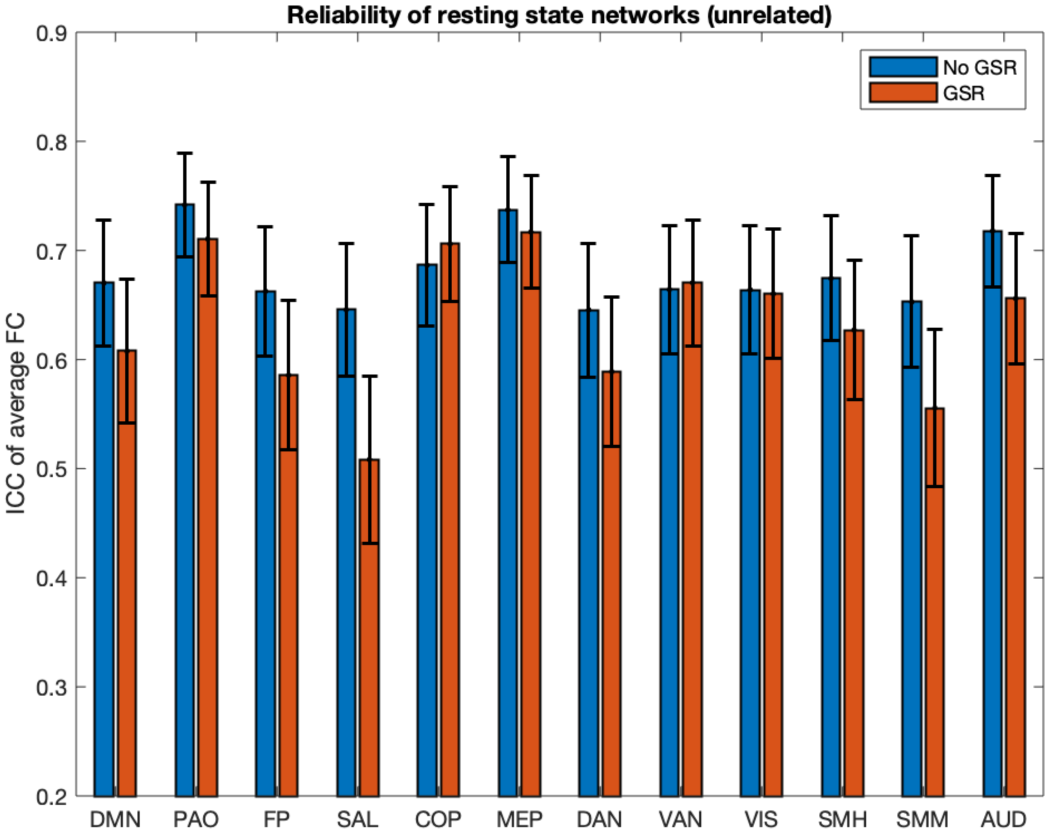


**Reliability of known resting state networks in unrelated participants.** We show the ICC and confidence intervals for the average connectivity within known resting state networks defined in accordance to (2). Abbreviations: ICC=intraclass correlation coefficient, GSR-=no global signal regression, GSR+=global signal regression, CI=confidence interval, DMN=default mode network, PAO=parieto-occipital, FP=fronto-parietal, SAL=salience, COP=cingulo-opercular, MEP=medial parietal, DAN=dorsal attention network, VAN=ventral attention network, VIS=visual, SMH=supplementary motor (hand), SMM=supplementary motor (mouth), AUD=auditory.

Figure S5

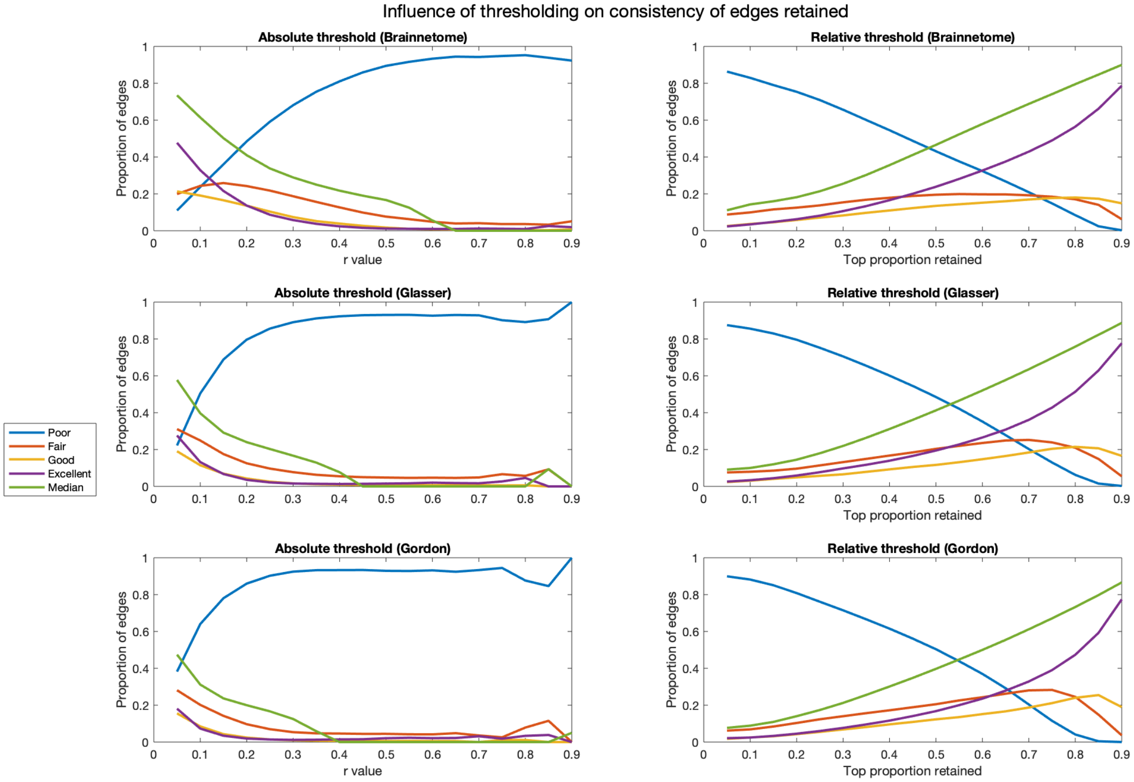


**Effects of thresholding on edge retention in unrelated participants.** In the Brainnetome, Glasser and Gordon atlases, for each absolute and relative threshold we show the proportion of edges that are consistently retained. As a measure of consistency, we use the number of participants in which the edge was retained at both timepoints divided by the ones in which it was retained at least once. For convenience, we then use the values defined in (1) to plot the ratio of edges having poor (ratio<0.40), fair (ratio=0.40-0.60), good (ratio=0.60-0.75) or excellent (ratio >0.75) consistency. For absolute thresholds (left) all edges below the value are set to 0, for relative ones (right) only the top percent corresponding to the threshold is retained. Abbreviations: r=Pearson correlation coefficient.

Figure S6

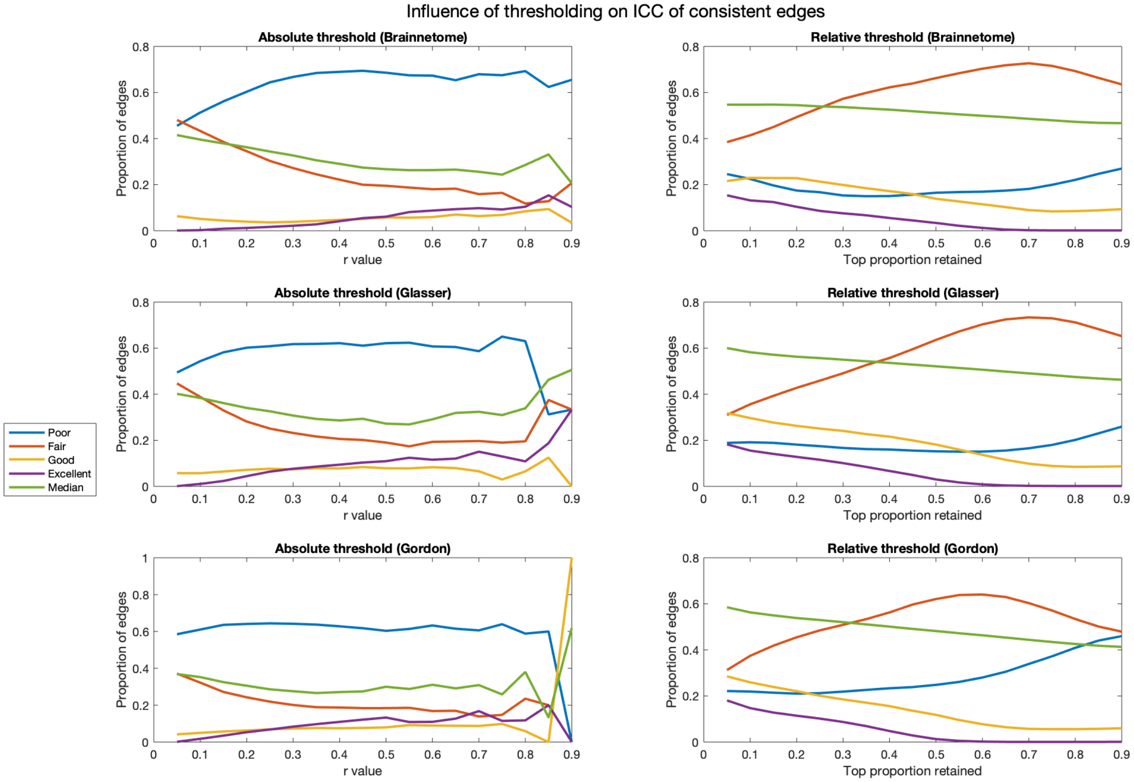


**Effects of thresholding on reliability in unrelated participants.** In the Brainnetome, Glasser and Gordon atlases, for each absolute and relative threshold we show the proportion of edges having poor (ICC<0.40), fair (ICC=0.40-0.60), good (ICC=0.60-0.75) or excellent (ICC>0.75) reliability. In this calculation, only subjects for which the edge was retained in both sessions were considered. For absolute thresholds (left) all edges below the value are set to 0, for relative ones (right) only the top percent corresponding to the threshold is retained. Abbreviations: r=Pearson correlation coefficient, ICC=intraclass correlation coefficient.

Figure S7

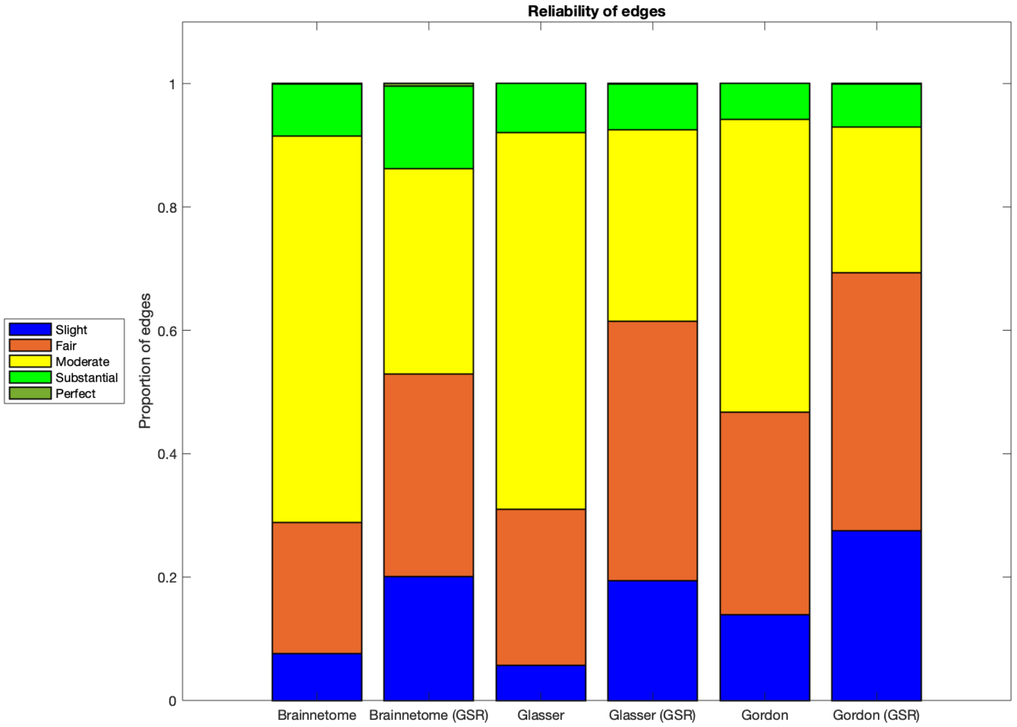


**Reliability of functional connectome edges using different ICC intervals.** For the Brainnetome, Glasser and Gordon atlases with and without performing GSR we show the proportion of edges having slight (<0.20), fair (0.20-0.40), moderate (0.40-0.60), substantial (0.60-0.80), and perfect (>0.80) in accordance to (3). Abbreviations: ICC=intraclass correlation coefficient, GSR-=no global signal regression, GSR+=global signal regression.

Figure S8

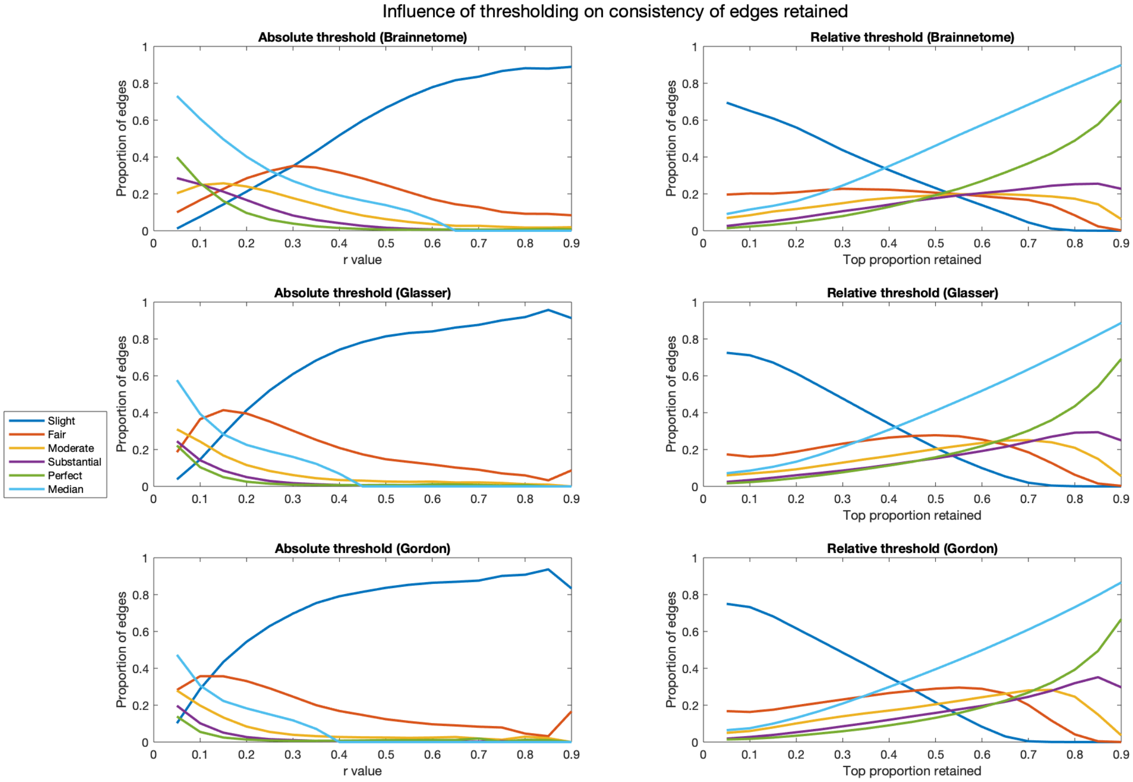


**Effects of thresholding on edge retention using different ICC intervals.** In the Brainnetome, Glasser and Gordon atlases, for each absolute and relative threshold we show the proportion of edges that are consistently retained. As a measure of consistency, we use the number of participants in which the edge was retained at both timepoints divided by the ones in which it was retained at least once. For convenience, we then use the values defined in (1) to plot the ratio of edges having slight (<0.20), fair (0.20-0.40), moderate (0.40-0.60), substantial (0.60-0.80), and perfect (>0.80) consistency. For absolute thresholds (left) all edges below the value are set to 0, for relative ones (right) only the top percent corresponding to the threshold is retained. Abbreviations: r=Pearson correlation coefficient.

Figure S9

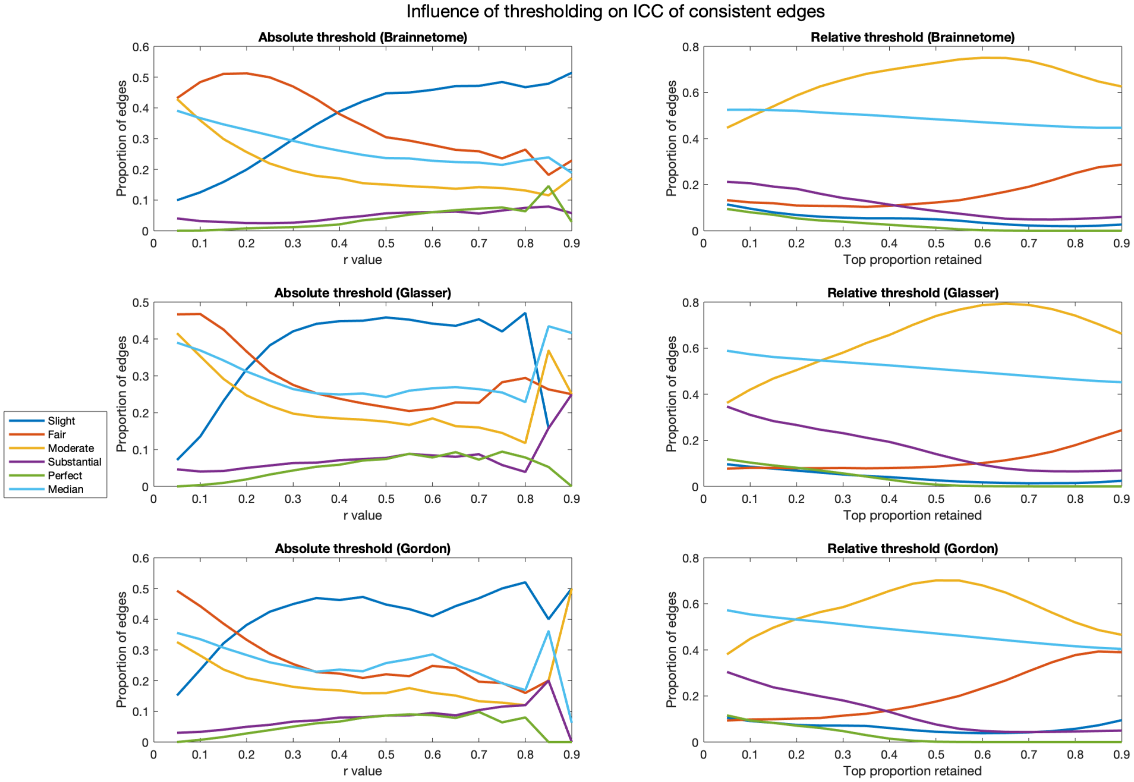


**Effects of thresholding on reliability using different ICC intervals.** In the Brainnetome, Glasser and Gordon atlases, for each absolute and relative threshold we show the proportion of edges having slight (<0.20), fair (0.20-0.40), moderate (0.40-0.60), substantial (0.60-0.80), and perfect (>0.80) reliability. In this calculation, only subjects for which the edge was retained in both sessions were considered. For absolute thresholds (left) all edges below the value are set to 0, for relative ones (right) only the top percent corresponding to the threshold is retained. Abbreviations: r=Pearson correlation coefficient, ICC=intraclass correlation coefficient

## References


1. D. V. Cicchetti, Guidelines, criteria, and rules of thumb for evaluating normed and standardized assessment instruments in psychology. *Psychological Assessment* **6**, 284–290 (1994).

2. E. M. Gordon, *et al.*, Generation and Evaluation of a Cortical Area Parcellation from Resting-State Correlations. *Cereb. Cortex* **26**, 288–303 (2016).

3. X.-X. Xing, X.-N. Zuo, The anatomy of reliability: a must read for future human brain mapping. *Sci. Bull.* **63**, 1606–1607 (2018).
